# Hypofractionated Radiotherapy Induces ISG15^+^MHC‐I^+^ Neutrophils to Augment Anti‐Tumor Immunity and Prime Immune Checkpoint Blockade Responses in Rectal Cancer

**DOI:** 10.1002/advs.202517450

**Published:** 2026-03-13

**Authors:** Lichao Liu, Haihong Wang, Mingjie Li, Qian Xu, Linlin Zheng, Chaoqun Han, Zhenwei Zou, Jinghua Ren, Xiaorong Dong, Peng Zhang, Kaixiong Tao, Zhenyu Lin, Tao Zhang

**Affiliations:** ^1^ Cancer Center Union Hospital Tongji Medical College Huazhong University of Science and Technology Wuhan China; ^2^ Institute of Radiation Oncology Union Hospital Tongji Medical College Huazhong University of Science and Technology Wuhan China; ^3^ Hubei Province Key Laboratory of Precision Radiation Oncology Wuhan China; ^4^ Department of Gastroenterology Union Hospital Tongji Medical College Huazhong University of Science and Technology Wuhan China; ^5^ Department of Gastrointestinal Surgery Union Hospital Tongji Medical College Huazhong University of Science and Technology Wuhan China; ^6^ The Eighth Hospital of Wuhan Wuhan China

**Keywords:** hypofractionated radiotherapy, immunotherapy, ISG15^+^MHC‐I^+^ neutrophils, LARC, NOD1 pathway

## Abstract

Our previous clinical trials had demonstrated that neoadjuvant hypofractionated radiotherapy (HFRT) combined with immunotherapy yields promising clinical outcomes in locally advanced rectal cancer (LARC). However, this combined modality benefits only a subset of patients, highlighting the need to uncover the mechanisms underlying how successful immunotherapy changes the tumor microenvironment to favor tumor control. Here, we showed that HFRT increases ISG15^+^MHC‐I^+^ neutrophil infiltration, which exhibits antigen‐presenting capabilities and is crucial for successful neoadjuvant therapy in rectal cancer. Mechanistically, HFRT promotes IFN‐α release, which activates the NOD1/NF‐κB pathway to drive MHC‐I expression in neutrophils. Adoptive transfer of ex vivo‐generated ISG15^+^MHC‐I^+^ neutrophils in mouse models enhanced intratumoral CD8^+^ T cell infiltration, synergizing with anti‐PD‐1 therapy to suppress tumor growth. This study uncovers an HFRT‐induced neutrophil subset that bridges local radiation with systemic immunity, providing a potential strategy to convert “cold” tumors to “hot” phenotypes for enhanced immunotherapy efficacy in microsatellite stability LARC.

## Graphical abstract

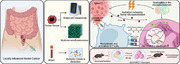

## 1 Introduction

The incidence of locally advanced rectal cancer (LARC) has been progressively increasing, posing growing therapeutic challenges in clinical oncology. The current standard‐of‐care involving neoadjuvant chemoradiotherapy followed by total mesorectal excision remains suboptimal, with persistent risks of distant metastasis and locoregional recurrence compromising long‐term survival outcomes [1, 2]. In the immunotherapy era, only ∼5% of LARC patients—those with microsatellite instability‐high (MSI‐H) or mismatch repair‐deficient (dMMR) tumors—respond well to immune checkpoint inhibitors [3, 4]. However, the remaining 95% of patients have a microsatellite stable (MSS) or mismatch repair‐proficient profile(pMMR), which are considered ‘cold’ tumors and render them less responsive to immunotherapy. Therefore, overcoming immunotherapy resistance in MSS/pMMR LARC remains an urgent unmet need.

Our group recently demonstrated in phase II and phase III clinical trials that a novel therapeutic strategy combining short‐course hypofractionated radiotherapy (5Gy×5) with sequential chemotherapy and immunotherapy significantly improves pathological complete response (pCR) rates [5, 6]. Our further research revealed that hypofractionated radiotherapy (HFRT) induces immunogenic cell death more effectively than conventional fractionation [7]. However, the precise mechanisms by which HFRT modulates the tumor microenvironment (TIME) remain incompletely understood [8, 9, 10].

Neutrophils, the most abundant circulating leukocytes, exhibit remarkable functional heterogeneity and plasticity within the TIME. Recent evidence shows their survival exceeds five days in circulation and is further extended in specialized microenvironments [11, 12, 13], enabling their differentiation into functionally distinct subsets. Although historically categorized into anti‐tumor (N1) and pro‐tumor (N2) phenotypes [14], single‐cell RNA sequencing studies have uncovered unprecedented heterogeneity of neutrophil populations [15, 16]. While neutrophils promote tumor progression through mechanisms such as extracellular matrix remodeling via MMP‐9 secretion and angiogenesis promotion through VEGF release [17], they also exert context‐dependent anti‐tumor effects through ROS‐mediated cytotoxicity [18] and immunomodulation (e.g., antigen cross‐presentation to dendritic cells and CXCL10‐dependent CD8^+^ T cell recruitment) [19, 20]. Recently, researchers have identified a distinct neutrophil population that acquires antigen‐presenting capabilities through leucine metabolic reprogramming, exhibiting broad‐spectrum anti‐tumor activity across multiple malignancies [21]. Emerging evidence suggests radiotherapy may serve as a powerful tool to modulate neutrophil function within the TIME [22]. Nevertheless, the changes of neutrophil subsets following radiotherapy remain uncharacterized and merit further investigation.

In this study, we employed single‐cell RNA sequencing to analyze rectal cancer tissues from patients receiving HFRT, with particular focus on radiation‐induced alterations in neutrophil populations. We identified a unique immunogenic neutrophil subset characterized by co‐expression of ISG15 and MHC class I molecules (ISG15^+^MHC‐I^+^ neutrophils). Mechanistically, HFRT induces the formation of ISG15^+^MHC‐I^+^ neutrophils and potentiates their antigen‐presenting function through the IFN‐α/NOD1 signaling axis. More importantly, adoptive transfer of HFRT‐primed ISG15^+^MHC‐I^+^ neutrophils significantly enhanced anti‐tumor T cell responses and potentiated immunotherapy efficacy in rectal cancer.

## 2 Result

### 2.1 Hypofractionated Radiotherapy Reprograms Neutrophil Subsets in LARC

To evaluate the impact of HFRT on the local immune microenvironment in LARC, this study prospectively collected paired pre‐neoadjuvant treatment(T1) biopsy samples and post‐neoadjuvant treatment(T3) surgical resection specimens from three patients undergoing combined HFRT and immunotherapy. Following standardized processing and quality control of raw sequencing data, 55,037 high‐quality cells were retained for downstream analysis. Sixteen distinct cell clusters were identified and annotated using standardized markers and gene expression profiles through unified, unsupervised, graph‐based clustering and visualization. These included CD4^+^ T cells, CD8^+^ T cells, Treg, Th17, NKT, MKI67^+^ T cells, neutrophils, B cells, Plasma, macrophages, monocytes, mast cells, and other immune subsets (Figure S1a,b,c). We next conducted differential abundance analysis using Milo, a statistical framework that performs difference inpresence tests by assigning cells to partially overlapping neighborhoods on a k‐NN graph (Figure S1d). Compared to baseline, post‐treatment samples exhibited decreased proportions of B cells, neutrophils, Tregs, Th17 cells, and proliferating T cells, alongside significant expansions of CD8^+^ T cells, plasma cells, and monocytes (Figure S1e). These findings indicate that HFRT induces comprehensive remodeling of the local immune landscape in LARC, characterized by cytotoxic cell enrichment and immunosuppressive population reduction.

**FIGURE 1:**
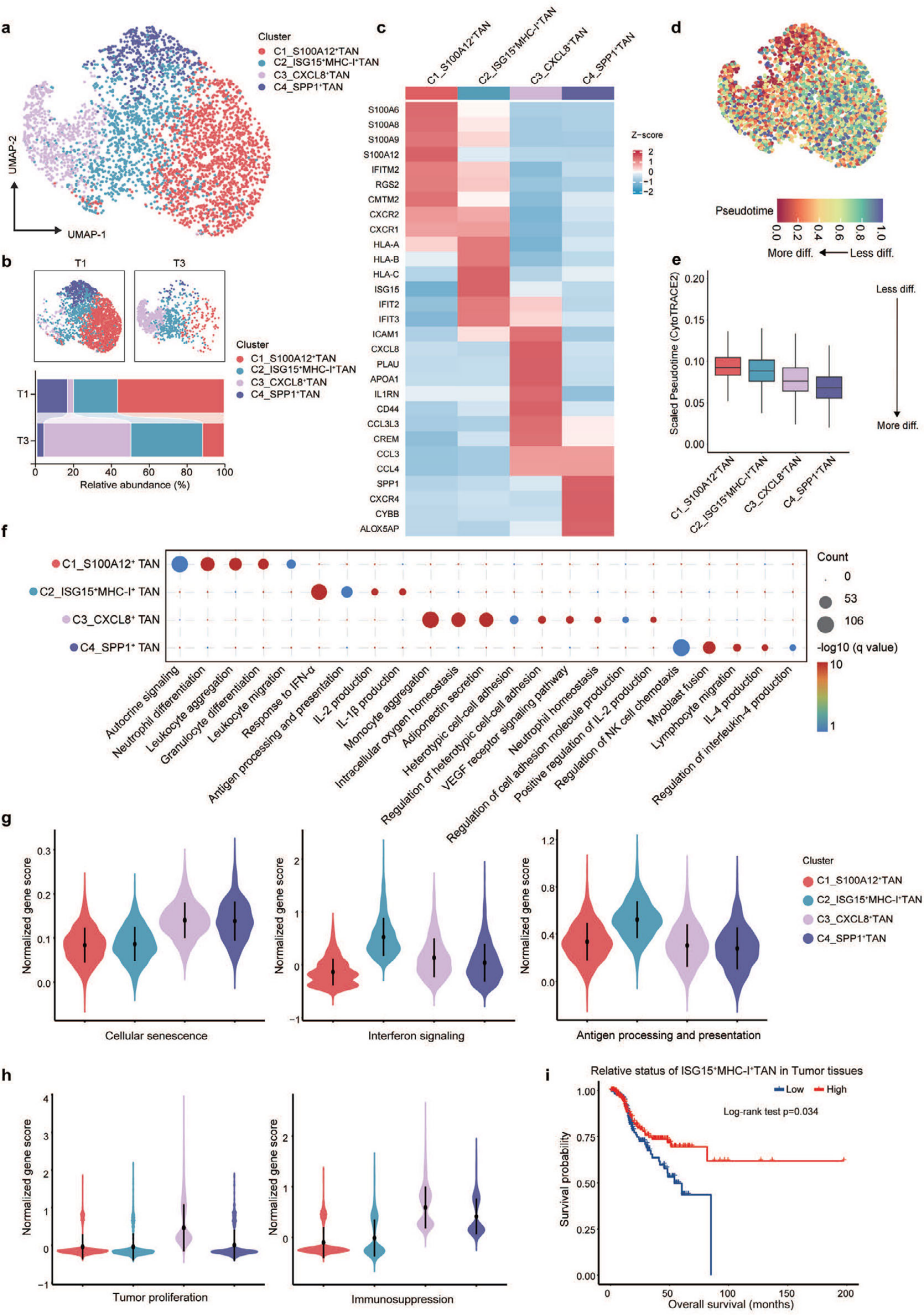
Hypofractionated Radiotherapy Reprograms Neutrophil Subsets in LARC. (a) Uniform manifold approximation and projection (UMAP) plot showing neutrophils clusters. Dots represent individual cells, and colors represent different cell subpopulations. (b) UMAP showing classification of neutrophils clusters in each condition (T1 vs. T3). Boxplots showing the alterations of cell clusters between T1 and T3. T1: pre‐neoadjuvant therapy. T3: post‐neoadjuvant therapy. (c) Heatmap showing the feature genes in neutrophils clusters from merged conditions. (d) Differentiation state estimated by CytoTRACE2. (e) Box plots showing the Pseudotime of different mono‐macrophage clusters. The black lines represent the developmental pathway estimated by Slingshot. (f) Dot plot showing the enriched GO pathways of differentially expressed genes for neutrophil clusters. Dot size represents gene counts in pathway and dot colour represents adjusted *p* value. (g) Expression of functional gene sets in neutrophil clusters before and after SIC by using AddModuleScore function of Seurat package. (h) Violin plots show the normalized gene set scores associated with tumor proliferation and immunosuppression in the four TAN subsets. (i) Kaplan–Meier curves for overall survival according to the relative status of ISG15^+^MHC‐I^+^ neutrophils in tumor tissues after radiation therapy in the TCGA database. Statistical analysis was performed using two‐sided log rank test.

The role of neutrophils in cancer immunology remains controversial, with evidence supporting both pro‐tumor and anti‐tumor functions. To elucidate changes in neutrophil subpopulations within the TIME following radiotherapy, we annotated cells based on published neutrophil subpopulation studies [23] and unique subsets identified in our sample. Neutrophils were categorized into four heterogeneous subpopulations based on their marker gene expression profiles: C1_S100A12^+^, C2_ISG15^+^MHC‐I^+^, C3_CXCL8^+^, and C4_SPP1^+^. C1_S100A12^+^ neutrophils exhibited high expression of immunosuppression‐related genes, including S100A12, S100A8, and S100A6. C2_ISG15^+^MHC‐I^+^ neutrophils were characterized by elevated levels of interferon‐responsive genes such as ISG15, HLA‐A, HLA‐B, and HLA‐C. C3_CXCL8^+^ neutrophils showed enrichment for genes associated with cytokine secretion, particularly CXCL8, PLAU, and APOA1. C4_SPP1^+^ neutrophils displayed high expression of immunosuppression‐linked markers, including SPP1, CXCR4, and CYBB (Figure 1a–c). We also conducted differential abundance analysis using Milo on a k‐NN graph. Compared to T1, the proportions of S100A12^+^ and SPP1^+^ neutrophils decreased, while CXCL8^+^ and ISG15^+^MHC‐I^+^ subsets significantly increased in T3(Figure S2a). To further verify the effect of HFRT on ISG15^+^MHC‐I^+^ neutrophils, we collected another LARC biopsy specimen from 12 patients before HFRT (T1) and after HFRT (T2) for scRNA‐seq, and performed subgroup analysis based on their treatment outcomes (Figure S2b,c). Differential abundance analysis was conducted using Milo on a k‐NN graph. CXCL8^+^, SPP1^+^, ISG15^+^MHC‐I^+^neutrophils were both increased after HFRT (Figure S2d), only ISG15^+^MHC‐I^+^neutrophils were increased in pCR patients (Figure S2e). Pseudotime trajectory analysis was performed on the identified subsets of neutrophils by CytoTRACE2.We ranked neutrophils according to their pseudotime, finding that C1_S100A12^+^ cells potentially remained at the initial stage, C2_ISG15^+^MHC‐I^+^, C3_CXCL8^+^ neutrophils remained at the intermediate stage, and C4_SPP1^+^ neutrophils remained at the terminus (Figure 1d,e). This pattern was corroborated by monocle3 and Slingshot (Figure S2f). Furthermore, we demonstrated the expression profiles of key genes in each neutrophil subset using a bubble plot (Figure S2g). Notably, MHC class II molecules, which are highly expressed in HLA‐DR^+^CD74^+^ neutrophils identified by Wu Y et al., were not highly expressed in ISG15^+^MHC‐I^+^ neutrophils; instead, MHC class I molecules were highly expressed [24]. This suggests that ISG15^+^MHC‐I^+^ neutrophils represent a unique subset that specifically responds to HFRT and concurrently highly expresses interferon‐stimulated genes and MHC class I molecules. Collectively, these results demonstrate that HFRT reshapes the neutrophil landscape in LARC, driving a shift from immunosuppressive subsets (S100A12^+^, SPP1^+^) toward interferon‐activated (ISG15^+^MHC‐I^+^) phenotypes, while pseudotime analysis suggests a dynamic progression from immature to terminally differentiated states.

### 2.2 ISG15^+^MHC‐I^+^ Neutrophils Exhibit Potent Immunostimulatory Capacity to Activate CD8^+^ T Cell Responses

To further characterize the biological states of the identified neutrophil clusters, we performed Gene Ontology pathway enrichment analysis to elucidate their functional properties (Figure 1f). Notably, the C2_ISG15^+^MHC‐I^+^ neutrophil subset exhibited enrichment in antigen processing and presentation and response to IFN‐α, suggesting potential anti‐tumor activity. Functional profiling using predefined gene sets—including cellular aging, interferon signaling, antigen presentation, tumor proliferation, and immune suppression—further supported these findings (Figure 1g,h, and Figure S3a). Specifically, the ISG15^+^MHC‐I^+^ neutrophils displayed reduced activity in pro‐tumor pathways but elevated interferon signaling, consistent with an immunostimulatory role. In contrast, the CXCL8^+^ neutrophils showed elevated activity in pro‐tumor functions such as angiogenesis and immunosuppression. Analysis of bulk RNA sequencing data from irradiated LARC tissues in the Cancer Genome Atlas (TCGA) database further revealed that high levels of ISG15^+^MHC‐I^+^ neutrophils are associated with better outcomes (Figure 1i), while CXCL8^+^ neutrophil abundance predicted poorer outcomes (Figure S3b). Additionally, the Human Protein Atlas database [25] corroborated that the ISG15 gene exhibits specific expression in granulocytes, notably in neutrophils and eosinophils (Figure S3c). Together, these results underscore the immune‐activating function of ISG15^+^MHC‐I^+^ neutrophils, prompting our subsequent focus on this key subpopulation for further investigation.

To elucidate the mechanism of ISG15‐mediated anti‐tumor effects, we first assessed its direct impact on tumor cells through a series of assays in vitro. Comprehensive evaluation, including cell viability, scratch wound healing, migration, and EDU‐based proliferation tests, unequivocally demonstrated that recombinant ISG15 protein exerted no detectable influence on tumor cell proliferation, motility, or invasive potential (Figure S4a–e), indicating that ISG15's anti‐tumor effects must be mediated through other mechanisms. We therefore investigated ISG15's immunomodulatory role using SARS‐CoV‐2 PLpro (SCP), a specific inhibitor that blocks ISG15 activity by cleaving its ubiquitin‐like modifications. In our subcutaneous tumor model, SCP administration significantly accelerated tumor progression (Figure S5a–d), indicating that ISGylation predominantly exerts regulatory effects by modulating immune cells within the tumor microenvironment, rather than directly interfering with tumor cell proliferation. Importantly, this pro‐tumor effect was completely abrogated when neutrophils were concurrently depleted using Anti‐Ly6G liposomes, demonstrating the essential role of neutrophils in mediating ISGylation's anti‐tumor activity. Flow cytometric analysis revealed that SCP treatment markedly reduced both total CD8^+^ T cells and IFNγ‐producing cytotoxic T cells within tumors (Figure S5e,f). Notably, this phenotype was neutrophil‐dependent, as concurrent neutrophil depletion restored CD8^+^ T cell parameters to baseline levels without affecting CD4^+^ T cell populations (Figure S5g). Given that the inhibition of ISGylation in vivo impaired the anti‐tumor activity of ISG15^+^MHC‐I^+^ neutrophils, we therefore further investigated ISGylation and its effect on MHC class I molecules in mouse primary neutrophils. Consistent with previous studies [26], ISGylation promoted MHC‐I expression, whereas SCP‐mediated inhibition of ISGylation abrogated this effect (Figure S5h), indicating the significant role of ISGylation in the development of ISG15^+^MHC‐I^+^ neutrophils. These findings delineate a novel immunoregulatory axis in which ISG15^+^MHC‐I^+^ neutrophils serve as pivotal orchestrators of anti‐tumor immunity. Rather than directly targeting tumor cells, these specialized neutrophils may mediate their protective effects through selective activation and maintenance of cytotoxic CD8^+^ T cell responses.

### 2.3 The Induction of ISG15^+^MHC‐I^+^ Neutrophils After Radiotherapy Was Dependent on IFN‐α

**FIGURE 2:**
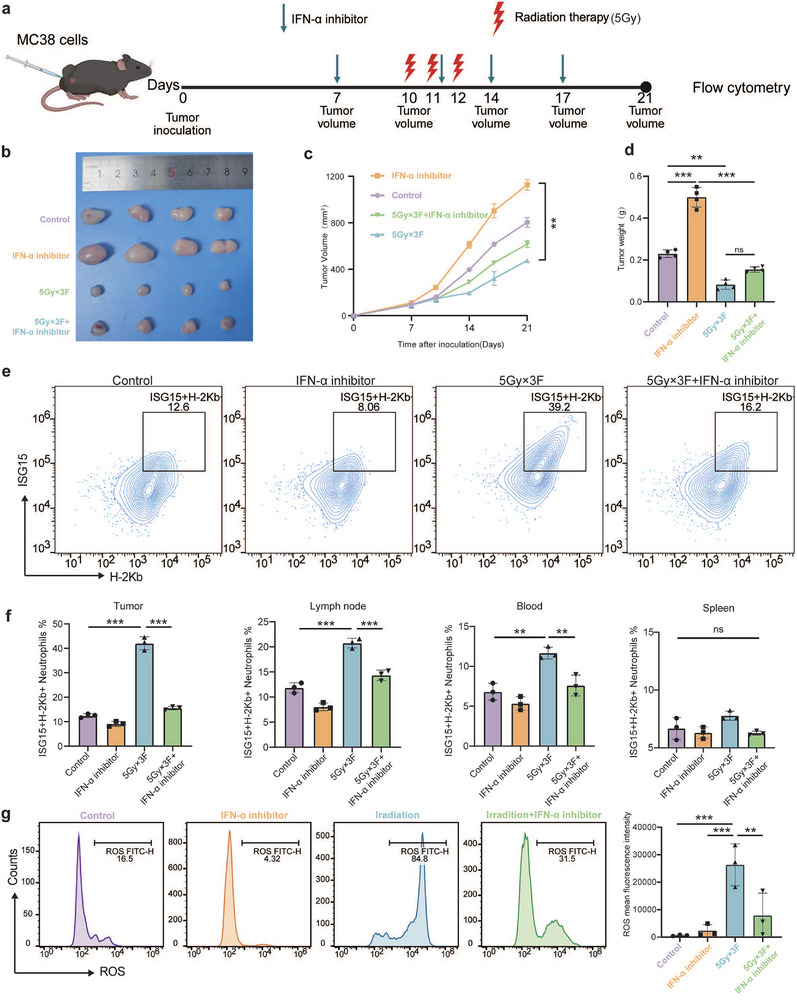
ISG15^+^MHC‐I^+^ Neutrophils Exhibit Potent Immunostimulatory Capacity to Activate CD8^+^ T Cell Responses. (a) Schematic diagram of MC38 subcutaneous tumor mouse model. C57BL/6 mice were subcutaneously inoculated with MC38 cell line (1×10^6^ cells/mouse) in the right lateral thigh. When tumors reached 100–150 mm^3^, mice were randomly assigned to four groups: Control, IFN‐α inhibitor,5Gy×3F Radiotherapy, and Combination group. Tumor growth was monitored (*n* = 6–8 per group). (b) Representative images of tumors in mice treated as described above. (c) Tumor growth of MC38 tumor‐bearing C57BL/6 mice treated with the indicated treatments (*n* = 4 per group). (d) Tumor weights in mice treated as described above. (*n* = 4 per group). One‐way ANOVA. (e) Representative flow contour plots of ISG15^+^H‐2Kb^+^ neutrophils in tumor tissue with different treatment assessed by flow cytometry (*n* = 3 per group). (f) Percentage of the ISG15^+^H‐2Kb^+^ neutrophil subset proportion in tumor, draining lymph node, peripheral blood, and spleen assessed with different treatment by flow cytometry (*n* = 3 per group). One‐way ANOVA. (g) Representative flow histograms (Left) and percentages (Right) of the release of ROS by ISG^+^MHC‐I^+^ neutrophils with different treatment assessed by flow cytometry (*n* = 3 per group). One‐way ANOVA. ∗*p *< 0.05; ∗∗*p* < 0.01; ∗∗∗*p* < 0.001; ∗∗∗∗*p* < 0.0001; ns, not significant.

We sought to elucidate the mechanisms underlying the generation of ISG15^+^MHC‐I^+^ neutrophils induced by hypofractionated radiotherapy (HFRT). Since ISG15 expression is known to be primarily regulated by interferon signaling, we first analyzed serum cytokine profiles from 20 LARC patients (10 with pCR; 10 non‐pCR) before and after HFRT. ELISA quantification showed a selective and statistically significant increase in IFN‐α levels in the pCR group after treatment, whereas IFN‐β and IFN‐γ levels remained unchanged (Figure S5i). To establish a causal relationship between IFN‐α signaling and the emergence of ISG15^+^MHC‐I^+^ neutrophils post‐HFRT, we employed a murine subcutaneous tumor model subjected to fractionated radiotherapy (5Gy×3 fractions) [7]. Radiotherapy significantly suppressed tumor growth; however, this antitumor effect was partially abolished by IFN‐α‐IFNAR‐IN‐1 hydrochloride, an inhibitor targeting the interaction between IFN‐α and its receptor IFNAR. (Figure 2a–d). Flow cytometric analysis of tumor‐infiltrating leukocytes revealed that radiotherapy robustly induced the accumulation of ISG15^+^MHC‐I^+^ neutrophils, and this enrichment was attenuated upon blockade of IFN‐α–IFNAR signaling (Figure 2e). Further analysis revealed that radiotherapy increased the frequency of ISG15^+^MHC‐I^+^ neutrophils in draining lymph nodes and peripheral blood, but only a marginal, non‐significant increase was observed in the spleen (Figure 2f). This pattern suggests that the expansion of these neutrophils is a systemic response mediated by radiotherapy‐induced IFN‐α production. To determine whether IFN‐α directly triggers this phenotypic change, we stimulated primary mouse neutrophils in vitro with conditioned medium from irradiated tumor cells (RTCM) or with IFN‐α. ISG15 expression was robustly induced, peaking after treatment with either RTCM (4 or 8 Gy) or with 10^4^ U/mL IFN‐α (Figure S5j,k). Taken together, these findings establish that the radiotherapy‐induced expansion of ISG15^+^MHC‐I^+^ neutrophils is dependent on IFN‐α signaling. We treated subcutaneous tumor‐bearing mice with a combination of 5 Gy×3 F radiotherapy and intraperitoneal injection of recombinant IFN‐α (Figure S6a,b), and further examined the infiltration status of ISG15^+^MHC‐I^+^ neutrophils across various organs (Figure S6c,d). We also examined the reactive oxygen species (ROS) production capacity of these neutrophils. As anticipated, irradiation significantly enhanced ROS generation, whereas inhibiting IFN‐α/IFNAR signaling—which blocks the differentiation of ISG15^+^MHC‐I^+^ neutrophils—substantially diminished ROS release (Figure 2g and Figure S6e). In summary, our findings demonstrate that HFRT activates the IFN‐α signaling pathway to promote the generation of ISG15^+^MHC‐I^+^ neutrophils, which in turn contribute to anti‐tumor immunity.

### 2.4 ISG15^+^MHC‐I^+^ Neutrophils Activate CD8^+^ T Cells Through Enhanced Function of Antigen‐Presenting

**FIGURE 3:**
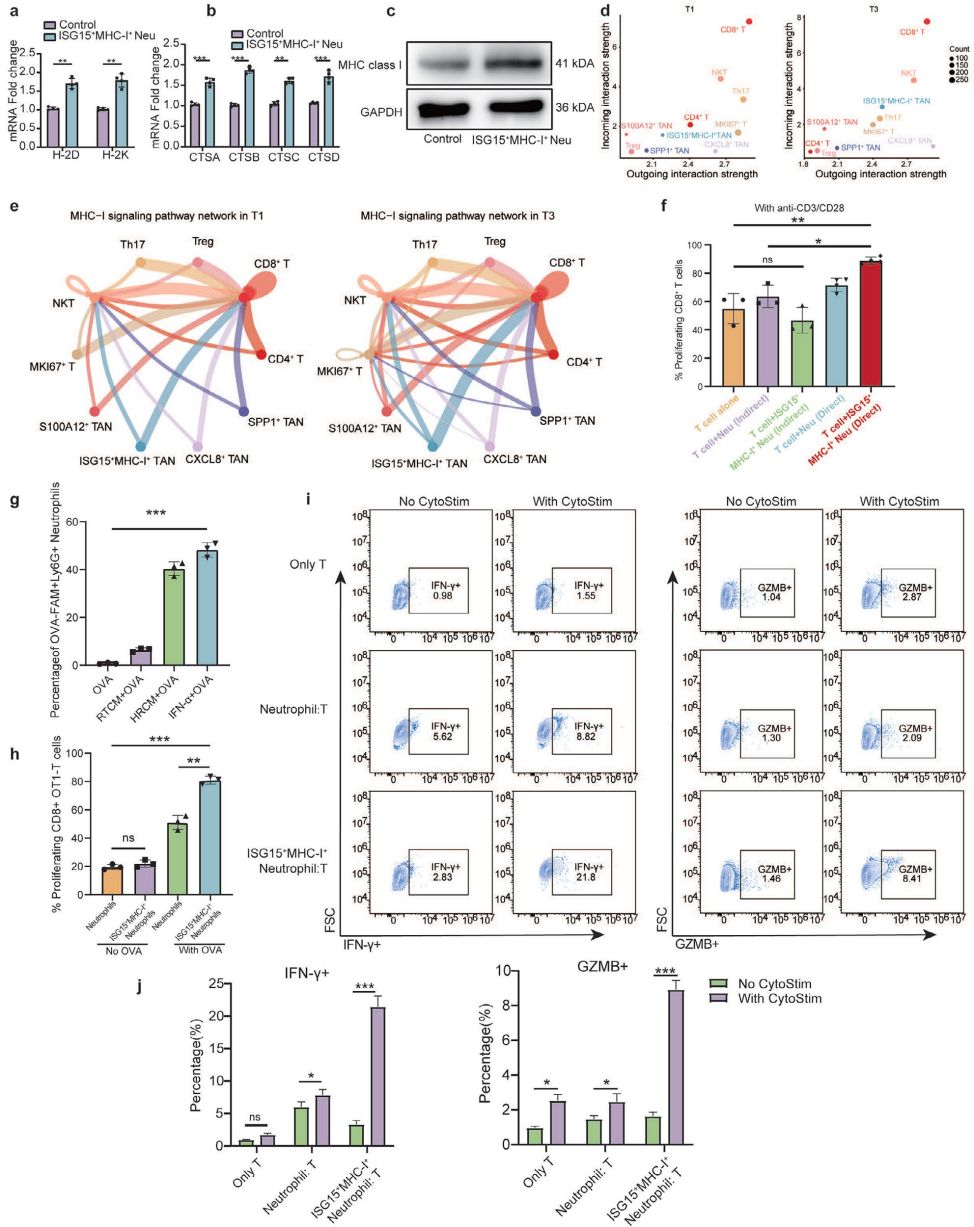
ISG15^+^MHC‐I^+^ neutrophils activate CD8^+^ T cells through enhanced function of antigen‐presenting. (a) Relative RNA expression of MHC‐I genes of HL60 with different treatment groups (*n* = 3 biological repeats). Two‐sided unpaired *t* test. (b) Relative RNA expression of antigen processing protease genes of the primary neutrophil with different treatment groups (*n* = 3 biological repeats). Statistical analysis was performed by using by two independent‐samples *t*‐test. (c) The expression of MHC class I of the primary neutrophil in protein level by using Western Blot. (d) Scatter plot of the incoming and outgoing interaction strength of each cell population in different conditions of LARC samples. (e) The inferred MHC‐I signaling networks in different conditions (T1 vs. T3). Circle sizes are proportional to the number of cells in each cell group, and edge width represents the communication probability. (f) Neutrophil treated with RT conditioned medium from MC38 cells were directly or indirect co‐cultured with primary CD8^+^ T cells. CD8^+^ T cell proliferation was measured by CFSE dilution. Percentages of proliferating CD8^+^ T cells (*n* = 3 per group). Two‐sided unpaired *t* test. (g) Neutrophils were stimulated with OVA‐FAM and conditioned media derived from MC38 cells exposed to either conventional‐dose irradiation (2 Gy) or hypofractionated‐dose irradiation (10 Gy). The proportion of OVA‐FAM^+^Ly6G^+^ neutrophils was quantified via flow cytometry (*n* = 3 per group). One‐way ANOVA. h. A co‐culture system of ISG15^+^MHC‐I^+^ neutrophils and CD8^+^ OT1 T cells (derived from OT1 mice) was established under conditions with or without OVA The proportion of CD8^+^ OT1 T cells in this system was quantified via flow cytometry (*n* = 3 per group). One‐way ANOVA. (i) and (j) MC38 naive CD8 + T cells were cultured in medium alone or cocultured with Neutrophils, IFNα‐treated Neutrophils, ± CytoSitm, at 2:1 ratio for 24 h. Representative flow contour plots of IFN‐γ^+^CD8^+^ T cells and GZMB^+^CD8^+^ T cells. Two‐sided unpaired *t* test. ∗*p *< 0.05; ∗∗*p* < 0.01; ∗∗∗*p* < 0.001; ∗∗∗∗*p* < 0.0001; ns, not significant.

Although historically regarded as weak antigen‐presenting cells, emerging evidence suggests neutrophils possess antigen‐presenting capabilities that may play a pivotal role in tumor immunity [21]. We assessed MHC‐I and antigen‐processing enzyme expression in ISG15^+^MHC‐I^+^ neutrophils in differentiated neutrophils derived from the human promyelocytic leukemia cell line HL60 educated by IFN‐α or conditioned medium of MC38 cells after radiotherapy. Compared to uninduced controls, ISG15^+^MHC‐I^+^ neutrophils demonstrated significant upregulation of MHC‐I and components of the antigen‐presentation machinery, as confirmed at both transcriptional (Figure 3a,b) and protein levels (Figure 3c). To characterize interactions between ISG15^+^MHC‐I^+^ neutrophils and other tumor‐infiltrating immune cells, we performed single‐cell RNA sequencing‐based interaction analysis. CD8^+^ T cells demonstrated the strongest bidirectional signaling with neutrophils, irrespective of treatment status. Notably, this communication was markedly enhanced after HFRT (Figure 3d). Ligand‐receptor analysis further identified robust interactions between neutrophil‐derived MHC‐I molecules and T cell‐expressed CD8α (Figure S7a), and pathway quantification confirmed an intensification of this MHC‐I‐dependent crosstalk post‐treatment (Figure 3e). Subsequently, we co‐cultured these in vitro‐induced ISG15^+^MHC‐I^+^ neutrophils with primary CD8^+^ T cells (Figure S7b). T cell proliferation was significantly activated only upon direct co‐culture (Figure 3f and Figure S7c), suggesting a contact‐dependent mechanism. To directly assess antigen‐processing capacity, neutrophils were pulsed with fluorescently labeled OVA protein. Induced ISG15^+^MHC‐I^+^ neutrophils exhibited significantly enhanced antigen uptake and presentation (Figure 3g and Figure S7d). Furthermore, coculture of OT1 mouse‐derived T cells with these neutrophils demonstrated that OT1‐T cells were significantly activated and proliferated by ISG15^+^MHC‐I^+^ neutrophils only in the presence of OVA, indicating that ISG15^+^MHC‐I^+^ neutrophils activate T cells via antigen presentation. (Figure 3h and Figure S7e). Furthermore, when co‐cultured with a general T cell stimulus (CytoStim), ISG15^+^MHC‐I^+^ neutrophils potently activated CD8^+^ T cells, inducing high expression of effector and cytotoxicity markers (Figure 3i,j), as previously described [27]. Finally, immunofluorescence analysis of post‐neoadjuvant LARC human specimens exhibited significantly greater co‐infiltration of ISG15^+^MHC‐I^+^ neutrophils and CD8^+^ T cells in patients achieving a pCR compared to non‐pCR patients (Figure S8a). Collectively, these findings establish that HFRT/IFN‐α reprograms neutrophils into proficient antigen‐presenting cells, thereby enhancing MHC‐I‐restricted antigen presentation and promoting productive interactions with CD8^+^ T cells.

### 2.5 IFN‐α Triggers NOD1/NF‐κB Signaling to Induce ISG15+MHC‐I+ Neutrophils Through MHC‐I Promoter Binding

**FIGURE 4:**
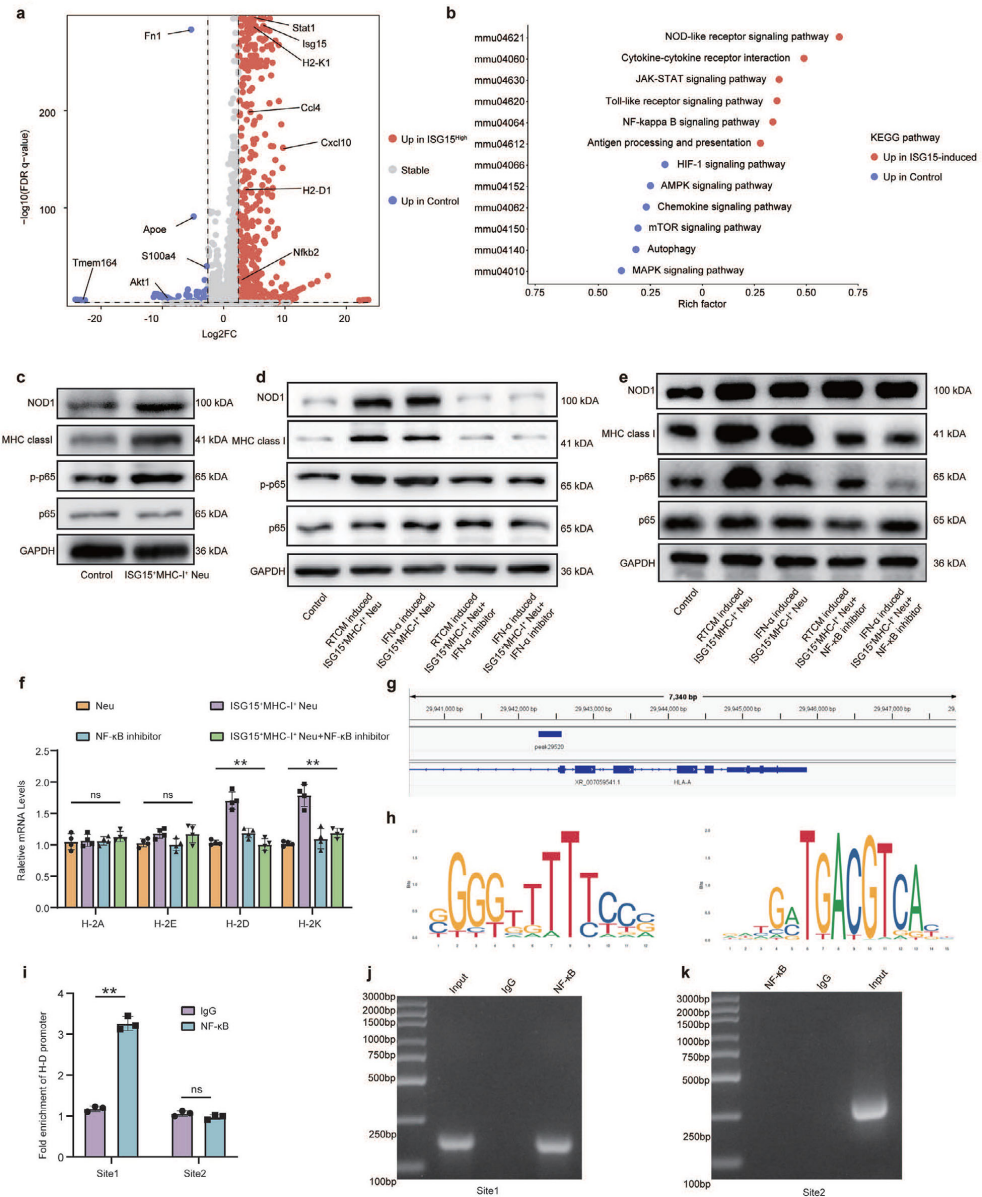
IFN‐α/NOD‐1/NF‐κB signaling Axis Enhances Antigen‐presenting Function of ISG15^+^MHC‐I^+^ Neutrophils via Direct MHC‐I Promoter Binding. (a) Volcano plot demonstrating differentially expressed genes between ISG15‐induced group and control group. Relevant genes were highlighted. (b) KEGG analysis of differentially expressed genes between ISG15‐induced group and control group. (c) Western blotting for the expression of MHC class I in the primary neutrophil and IFN‐α induced ISG15^+^MHC‐I^+^ neutrophils. (d) Western blotting for the expression of MHC class I, NOD1, p‐p65, and p65 in the primary neutrophil, ±IFN‐α‐IFNAR‐IN‐1 hydrochloride. (e) Western blotting for the expression of MHC class I, NOD1, p‐p65, and p65 in the primary neutrophil, ±NLRP3‐IN‐32. (f) Relative RNA expression of MHC‐I and MHC‐II of the primary neutrophil with different treatment groups (*n* = 3 biological repeats). One‐way ANOVA. (g) The ChIP‐seq of NF‐κB on the promoter region of *MHC‐I*. (h) Prediction of NF‐κB binding sites in the MHC‐I promoter region using the JASPAR database. (i) The ChIP‐qPCR of NF‐κB on the promoter region of *MHC‐I* in HL60 cells. Two‐sided unpaired *t*‐test. (j) and (k). The DNA electrophoresis of the products from the ChIP assay. ∗*p *< 0.05; ∗∗*p* < 0.01; ∗∗∗*p* < 0.001; ∗∗∗∗*p* < 0.0001; ns, not significant.

To elucidate the pathway by which HFRT or IFN‑α mediates phenotypic remodeling of neutrophils, we performed transcriptome sequencing on radiation‑conditioned medium (RTCM)‐induced ISG15^+^MHC‑I^+^ neutrophils. Compared with controls, these neutrophils displayed 2841 upregulated and 1,921 downregulated transcripts (FDR < 0.05, |log_2_FC| > 1; Figure 4a). KEGG pathway enrichment analysis indicated that the NOD1, JAK, and NF‑κB signaling pathways were all upregulated in ISG15^+^MHC‑I^+^ neutrophils (Figure 4b). Corroborating these transcriptional findings, Western blot analysis confirmed markedly elevated protein levels of NOD1, phospho‑p65 (Ser536), and JAK1 in this neutrophil subset (Figure 4c and Figure S8b). Treatment with a selective IFN‑α/IFNAR inhibitor effectively suppressed the upregulation of NOD1, phospho‑p65, and MHC‑I in RTCM‑ or IFN‑α‑stimulated ISG15^+^MHC‑I^+^ neutrophils (Figure 4d). Subsequent targeted inhibition of NOD1 similarly reduced MHC‑I and phospho‑p65 expression in these neutrophils, whereas JAK1 inhibition showed no comparable effect (Figure S8c), consistent with a model in which IFN‑α modulates downstream NF‑κB activity and MHC‑I expression primarily through NOD1‑dependent signaling rather than via JAK1. Moreover, pharmacological inhibition of NF‐κB signaling using NLRP3‐IN‐32 markedly decreased phospho‐p65 levels and, correspondingly, MHC‐I expression, while NOD1 expression remained unchanged, further confirming the upstream role of NOD1 in this axis (Figure 4e). Correspondingly, NLRP3‐IN‐32 treatment also reduced MHC‐I mRNA levels in primary neutrophils (Figure 4f). In line with the in vitro data, the NOD1 inhibitor substantially attenuated the anti‐tumor efficacy of HFRT in vivo (Figure S8d,e). Flow cytometry further demonstrated that NOD1 inhibitor treatment completely abrogated HFRT‐driven intra‐tumoral expansion of ISG15^+^MHC‐I^+^ neutrophils (Figure S8f,g). Bioinformatic analysis of the MHC‐I promoter revealed an NF‐κB binding peak at −29 520 bp (Figure 4g) encompassing two predicted consensus sequences (Figure 4h). ChIP‐qPCR confirmed specific NF‐κB binding to Site 1, but not to Site 2 (Figure 4i–k), thus establishing a direct mechanism by which NF‑κB upregulates MHC‑I transcription in neutrophils. In summary, these findings establish that high‐dose radiation drives the generation of ISG15^+^MHC‐I^+^ neutrophils via activation of the IFN‐α/NOD1/NF‐κB signaling cascade.

### 2.6 Adoptive Transfer of ISG15^+^MHC‐I^+^ Neutrophils Suppresses Tumor Growth and Exerts a Synergistic Effect with Immunotherapy

**FIGURE 5:**
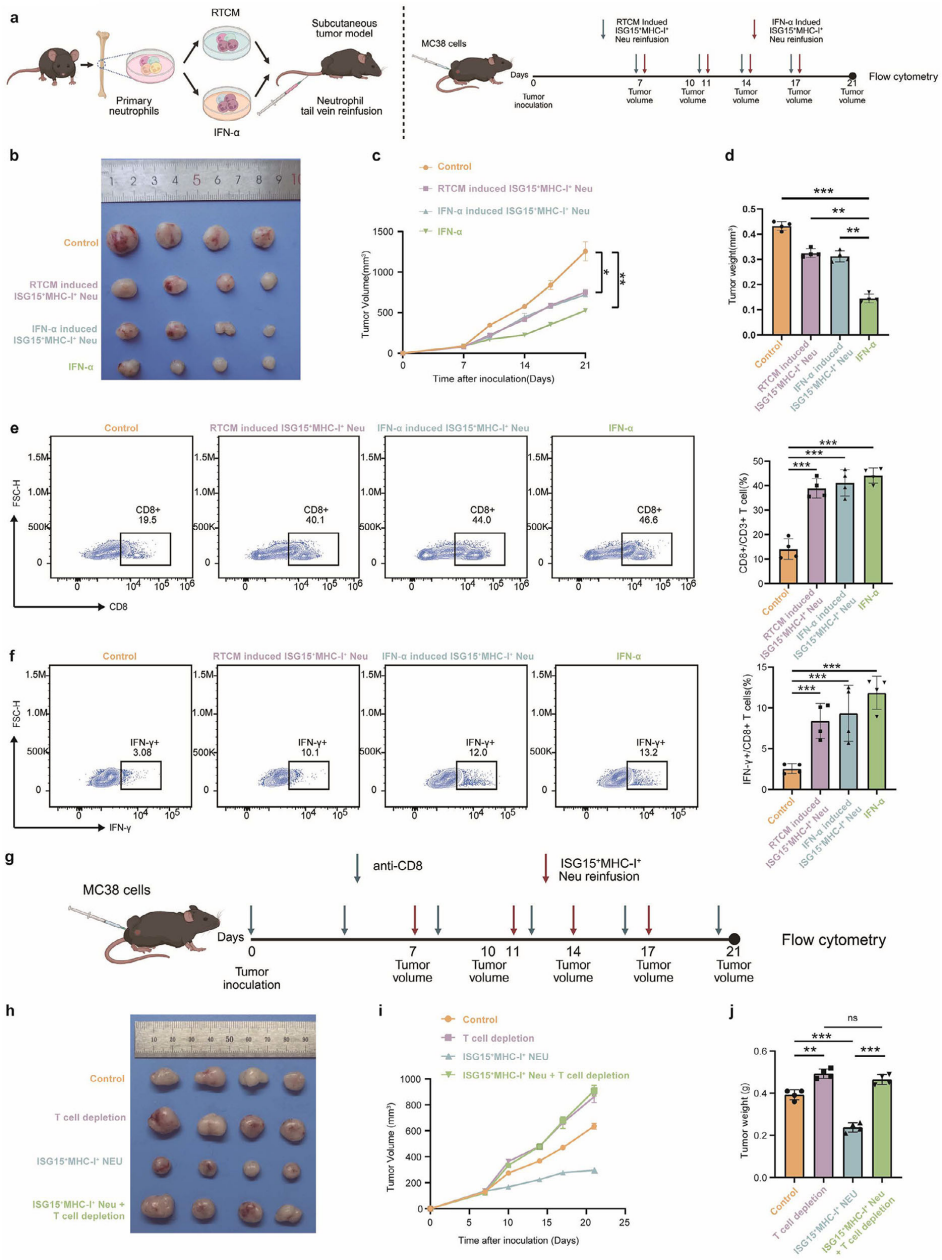
Adoptive Transfer of ISG15^+^MHC‐I^+^ neutrophils Suppresses Tumor Growth In Vivo. (a) Schematic diagram of the MC38 subcutaneous tumor mouse model. C57BL/6 mice were subcutaneously inoculated with MC38 cell line (1×10^6^ cells/mouse) in the right lateral thigh. When tumors reached 100–150 mm^3^, mice were randomly assigned to four groups: Control, RTCM induced ISG15^+^MHC‐I^+^ neutrophil reinfusion, IFN‐α induced ISG15^+^MHC‐I^+^ neutrophil reinfusion, and IFN‐α group. Tumor growth was monitored. (b) Representative images of tumors in mice treated as described above. (c) Tumor growth of MC38 tumor‐bearing C57BL/6 mice treated with the indicated treatments. (d) Tumor weights in mice treated as described above. One‐way ANOVA. (e) Representative flow plot (left) and percentages (right) of CD8^+^ T cells (CD8^+^/CD3^+^ T cells) in tumor tissue (*n* = 3 per group). One‐way ANOVA. (f) Representative flow plot (left) and percentages (right) of IFN‐γ^+^CD8^+^ T cells (IFN‐γ^+^/CD8^+^ T cells) in tumor tissue (*n* = 3 per group). One‐way ANOVA. (g) Schematic diagram of MC38 subcutaneous tumor mouse model. C57BL/6 mice were subcutaneously inoculated with MC38 cell line (1×10^6^ cells/mouse) in the right lateral thigh. When tumors reached 100–150 mm^3^, mice were randomly assigned to four groups: Control, T cell depletion, ISG15^+^MHC‐I^+^ neutrophil reinfusion, and Combination group. Tumor growth was monitored. (h) Representative images of tumors in mice treated as described above. (i) Tumor growth of MC38 tumor‐bearing C57BL/6 mice treated with the indicated treatments (*n* = 4 per group). (j) Tumor weights in mice treated as described above (*n* = 4 per group). One‐way ANOVA. ∗*p *< 0.05; ∗∗*p* < 0.01; ∗∗∗*p* < 0.001; ∗∗∗∗*p* < 0.0001; ns, not significant.

**FIGURE 6:**
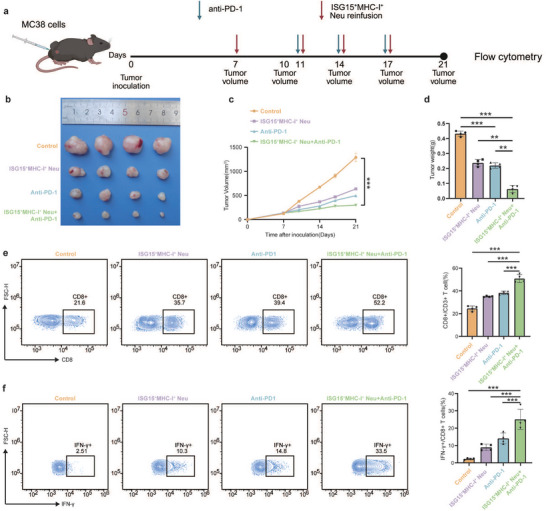
Adoptive Transfer of ISG15^+^MHC‐I^+^ Neutrophils Exerts Synergistic Antitumor Effects with Immunotherapy. (a) Schematic diagram of MC38 subcutaneous tumor mouse model. C57BL/6 mice were subcutaneously inoculated with MC38 cell line (1×10^6^ cells/mouse) in the right lateral thigh. When tumors reached 100–150 mm^3^, mice were randomly assigned to four groups: Control, ISG15^+^MHC‐I^+^ neutrophil reinfusion, Anti‐PD‐1, and Combination group. Tumor growth was monitored (*n *= 6–8 per group). (b) Representative images of tumors in mice treated as described above. (c) Tumor growth of the MC38 tumor‐bearing C57BL/6 mice treated with the indicated treatments. (d) Tumor weights in mice treated as described above. One‐way ANOVA. (e) Representative flow plot (left) and percentages (right) of CD8^+^ T cells (CD8^+^/CD3^+^ T cells) in tumor tissue (*n* = 3 per group). One‐way ANOVA. (f) Representative flow plot (left) and percentages (right) of IFN‐γ^+^CD8^+^ T cells (IFN‐γ^+^/CD8^+^ T cells) in tumor tissue (*n* = 3 per group). One‐way ANOVA. ∗*p *< 0.05; ∗∗*p* < 0.01; ∗∗∗*p* < 0.001; ∗∗∗∗*p* < 0.0001; ns, not significant.

Next, we investigated the delivery of ISG15^+^MHC‐I^+^ neutrophils into the TME as another therapeutic option. We isolated mouse bone neutrophils, stimulated them using radiation‐conditioned medium (RTCM) or IFN‐α, and transferred them into the subcutaneous tumor‐bearing mouse via the tail vein (Figure 5a). As shown in Figure 5b–d, adoptive transfer of ISG15^+^MHC‐I^+^ neutrophil significantly inhibited tumor growth, though less potently than intraperitoneal IFN‐α monotherapy. Flow cytometry revealed that the adoptive transfer of ISG15^+^MHC‐I^+^ neutrophils significantly augmented intratumoral CD8^+^ T cells infiltration, without significantly affecting CD4^+^ T cells (Figure 5e and Figure S9a). Furthermore, transferred neutrophils enhanced the frequency of CD8^+^IFN‐γ^+^ T cells (Figure 5f), indicating augmented T cell effector function. To establish T cell dependence for the observed antitumor effects, we performed CD8^+^ T cell depletion before neutrophil transfer (Figure 5g). T cell depletion completely abrogated the tumor‐suppressive efficacy of adoptively transferred ISG15^+^MHC‐I^+^ neutrophils (Figure 5h–j), demonstrating their antitumor activity requires intact T cell‐mediated immunity. To further investigate the infiltration profile of neutrophils in other organs following adoptive transfer of ISG15^+^MHC‐I^+^ neutrophils, we established an MC38‐OVA subcutaneous xenograft model using OT1 mice. Consistent with the above findings, adoptive transfer of ISG15^+^MHC‐I^+^ neutrophils resulted in marked suppression of tumor growth (Figure S9b,c). Concomitantly, ISG15^+^H‐2Kb‐SIINFEKL^+^ neutrophils and CD8^+^ T cells were markedly enriched in the tumor, peripheral blood, and draining lymph nodes, indicating that ISG15^+^MHC‐I^+^ neutrophils process OVA released from lysed tumor cells, express H‐2Kb‐SIINFEKL complexes on their surface, and thereby potentiate OT1‐T cell activation (Figure S9d,e). This finding further confirms the antigen‐presenting properties of ISG15^+^MHC‐I^+^ neutrophils. We subsequently evaluated the synergistic potential of combining adoptive transfer of these neutrophils with anti‐PD‐1 therapy in a subcutaneous tumor mouse model (Figure 6a). Compared to either treatment alone, the combination therapy showed a synergistic inhibitory effect on tumor growth (Figure 6b–d). Flow cytometry analysis revealed that adoptive transfer of ISG15^+^MHC‐I^+^ neutrophils enhanced intratumoral infiltration of CD8^+^ T cells, albeit to a lesser extent than anti‐PD‐1 monotherapy (Figure 6e). As expected, the combination treatment synergistically increased CD8^+^ T cell infiltration within tumors. A corresponding enhancement was also observed in IFN‐γ^+^ CD8^+^ T cells (Figure 6f), while CD4^+^ T cell infiltration remained unaltered (Figure S9f). Together, these results confirm a synergistic interaction between adoptive transfer of ISG15^+^MHC‐I^+^ neutrophils and immune checkpoint blockade.

### 2.7 Elevated ISG15^+^MHC‐I^+^ Neutrophils Infiltration Associated With Neoadjuvant Therapy Efficacy in LARC

**FIGURE 7:**
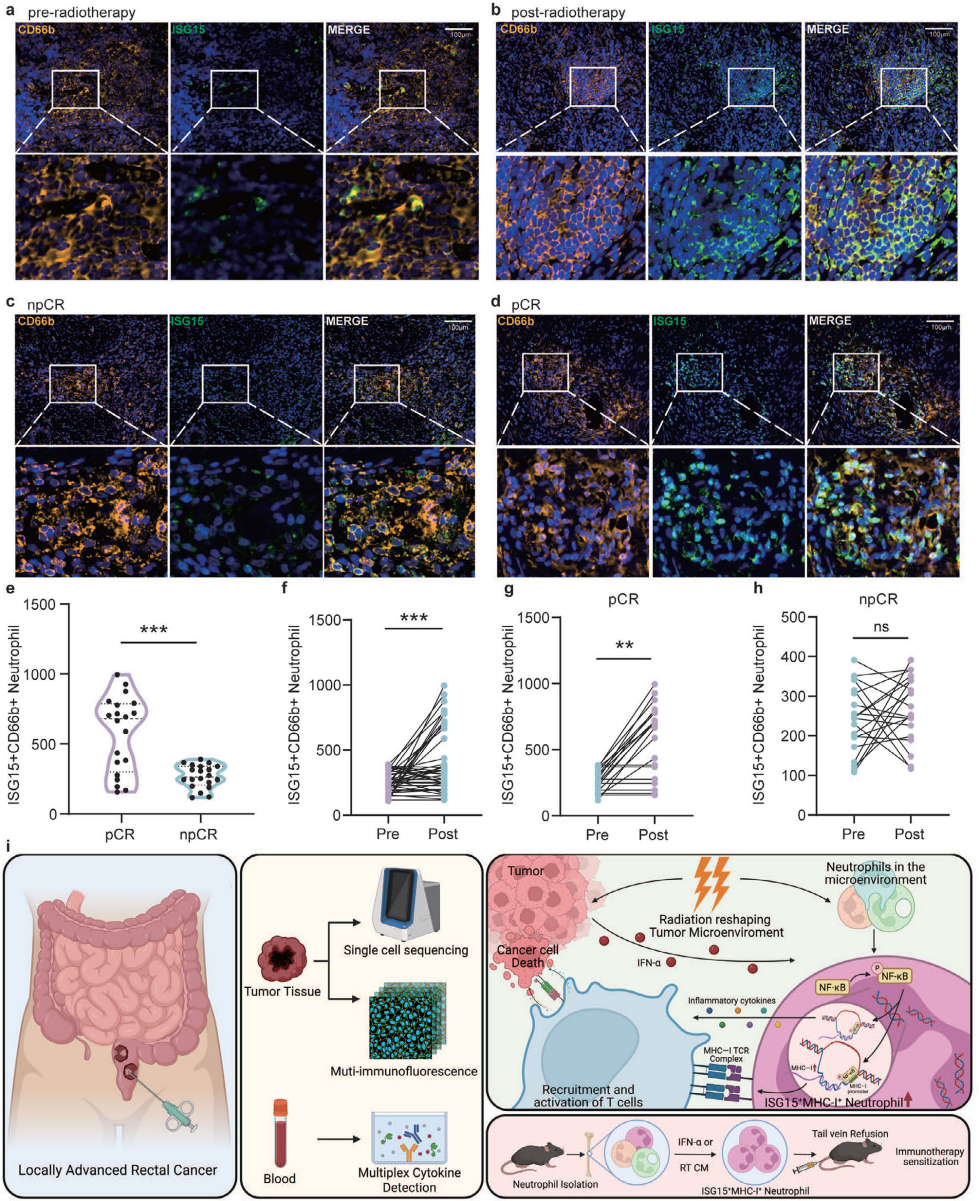
Elevated ISG15^+^MHC‐I^+^ Neutrophils infiltration associated with Neoadjuvant Therapy Efficacy in LARC. (a–d) Representative mIHC images of ISG15^+^MHC‐I^+^ neutrophils in the post‐ or pre‐neoadjuvant therapy tumor sample of npCR or pCR patient of LARC. Scale bars 100 µm (upper) and 20 µm (lower). (e) The difference in the density of ISG15^+^MHC‐I^+^ neutrophils from post‐neoadjuvant therapy between pCR (*n* = 30) and npCR patients (*n* = 30) via mIHC assay. Two‐sided unpaired *t* test. (f) The temporal alterations of the density of ISG15^+^MHC‐I^+^ neutrophils from pre‐ and post‐ neoadjuvant therapy in LARC patients (*n* = 60) via mIHC assay. Two‐sided paired *t* test. (g) The temporal alterations of the density of ISG15^+^MHC‐I^+^ neutrophils from pre‐ and post‐ neoadjuvant therapy in pCR patients (*n* = 30) via mIHC assay. Two‐sided paired *t* test. (h) The temporal alterations of the density of ISG15^+^MHC‐I^+^ neutrophils from pre‐ and post‐ neoadjuvant therapy in npCR patients (*n* = 30) via mIHC assay. Two‐sided paired *t* test. (i) The schematic diagram of the whole article. ∗*p *< 0.05; ∗∗*p* < 0.01; ∗∗∗*p* < 0.001; ∗∗∗∗*p* < 0.0001; ns, not significant.

To determine the clinical relevance of ISG15^+^MHC‐I^+^ neutrophils in locally advanced rectal cancer, we established a retrospective cohort of 40 patients, 20 of whom (50%) achieved pCR (Table S1). Except for magnetic resonance tumor regression grade (mrTRG), the distribution of other major clinical characteristics did not differ significantly between the pCR and non‐pCR groups. By comparing multiplex immunohistochemistry results from tumor tissue samples before and after neoadjuvant therapy, we observed a significant increase in ISG15^+^MHC‐I^+^ neutrophil density following treatment, with higher density in pCR patients compared to non‐pCR patients (Figure 7a–f). Further analysis revealed that the density of ISG15^+^MHC‐I^+^ neutrophils within tumors increased significantly only in pCR patients after neoadjuvant therapy, with no notable change observed in non‐pCR patients (Figure 7g,h). This finding was independently validated by flow cytometry analysis of peripheral blood samples, which yielded consistent results with the tissue‐level assays (Figure S9g). Furthermore, multivariate Cox regression analysis revealed that elevated levels of ISG15^+^MHC‐I^+^ neutrophils in peripheral blood after HFRT were significantly correlated with an increased probability of attaining pCR (Table S2). In summary, ISG15^+^MHC‐I^+^ neutrophils may serve as a potential biomarker for predicting responses to radiotherapy and immunotherapy in patients with locally advanced rectal cancer.

## 3 Discussion

Recent studies suggest that HFRT not only exerts direct cytotoxic effects but also modulates the tumor immune microenvironment. In particular, neutrophils—traditionally viewed as short‐lived innate immune cells—have emerged as key players in HFRT‐induced immune remodeling. Our research explores a novel mechanism by which HFRT activates neutrophils, driving the differentiation of a distinct immunosuppressive subset into an immunostimulatory phenotype. This reprogramming enhances antigen presentation and T‐cell priming, thereby overcoming the inherent resistance of MSS rectal cancer to immune checkpoint blockade. By elucidating the crosstalk between HFRT, neutrophil polarization, and adaptive immunity, this study provides a rationale for combining HFRT with immunotherapy in MSS rectal cancer, offering a promising strategy to expand the clinical benefits of immunotherapy to a broader patient population.

The functional dichotomy of neutrophils into pro‐tumor (N2) and anti‐tumor (N1) subsets, as initially proposed by Fridlender et al., has provided a foundational framework for understanding neutrophil plasticity in cancer [14]. However, this binary classification inadequately captures the full spectrum of neutrophil heterogeneity, particularly in the context of dynamic therapeutic interventions. Recent advances in single‐cell sequencing have unveiled a spectrum of neutrophil subsets with distinct functional profiles [23, 28]. For instance, in liver tumor, Xue R et al. identified 11 neutrophil clusters, including the immunosuppressive Neu_09_IFIT1 subset characterized by high PD‐L1 expression [23]. Similarly, in lung cancer, Sell^High^ neutrophils were found to accumulate in immunotherapy‐responsive tumors, correlating with interferon‐stimulated gene signatures. A pan‐cancer study further identified HLA‐DR^+^CD74^+^ neutrophils with enhanced antigen‐presenting capacity, driven by leucine metabolism [21]. These findings underscore the critical role of microenvironmental cues in shaping neutrophil diversity. In our study, single‐cell analysis of rectal cancer tissues following HFRT uncovered four distinct neutrophil subpopulations: S100A12^+^, ISG15^+^MHC‐I^+^, CXCL8^+^, and SPP1^+^ subsets. The ISG15^+^MHC‐I^+^ subset exhibited immunostimulatory properties, marked by MHC‐I upregulation and interferon‐response pathway activation. In contrast, the CXCL8^+^ subset displayed immunosuppressive characteristics. Notably, while ISG15^+^MHC‐I^+^ neutrophils in our study share transcriptional similarities with the ISG15^+^IFIT1^+^ subset (reported by Wu et al. [21]. to express PD‐L1), we observed no PD‐L1 upregulation in our cohort. This discrepancy may stem from differences in cancer type or therapeutic context, as our cohort received radiotherapy—a potent modulator of the tumor microenvironment. Radiotherapy‐induced IFN‐α likely drives the differentiation of ISG15^+^MHC‐I^+^ neutrophils with unique immunogenic features, diverging from the PD‐L1‐dependent suppression observed in untreated pan‐cancer models. These findings emphasize the need to investigate neutrophil plasticity under specific treatment conditions, as therapeutic interventions may redefine subset functionalities. Future studies should explore the temporal dynamics of neutrophil subpopulations during combined HFRT and immunotherapy, addressing whether ISG15^+^MHC‐I^+^ neutrophils serve as universal biomarkers or context‐dependent effectors across cancer types.

Emerging evidence suggests that radiation therapy recruits neutrophils to irradiated sites, likely mediated by the release of radiation‐induced damage‐associated molecular patterns (DAMPs) [29]. Furthermore, radiation has been shown to modulate neutrophil activation states, triggering the secretion of pro‐inflammatory cytokines [30]. In this study, we found that HFRT induces neutrophil release of ROS, and ISG15^+^MHC‐I^+^ neutrophils have stronger ROS release function. While neutrophils have traditionally been regarded as weak antigen‐presenting cells, a recent study has redefined their immunologic role, revealing mechanisms such as citrullination that augment their antigen‐presenting capacity [21]. Our research further confirms the role of radiation therapy in enhancing neutrophil antigen presentation function. We found that radiation therapy triggers IFN‐α production, leading to an increase in ISG15^+^MHC‐I^+^ neutrophils with an antigen‐presenting phenotype. This discovery is not only consistent with the new perspective on the antigen presentation function of neutrophils, but also makes neutrophils a bridge for communicating the synergistic effects of radiation therapy and immunotherapy. As this study was conducted in the context of HFRT, we found through induction of gradient radiotherapy dose conditioned medium that 4 Gy or 8 Gy irradiation in vitro can lead to maximum ISG15^+^MHC‐I^+^ neutrophil induction. On the one hand, this confirms the mechanism of HFRT at the cellular level, and at the same time, it prompts us to deeply consider the application of radiotherapy as a local treatment and systemic immune regulation method.

Previous studies have demonstrated that type I interferon can upregulate MHC class I molecule expression in human monocytes and endothelial cells [31, 32, 33]. However, the precise regulatory mechanism in neutrophils remains poorly understood. Interestingly, IFN‐γ, another interferon family member, is known to enhance MHC class I expression through the JAK‐STAT signaling pathway [34]. Our transcriptome sequencing analysis reveals a novel finding: the NF‐κB signaling pathway appears to play a pivotal role in IFN‐α‐mediated MHC‐I upregulation in neutrophils. Through chromatin immunoprecipitation (ChIP) assays and binding site prediction analyses, we identified that nuclear‐translocated NF‐κB can bind to predicted binding site 1 in the MHC‐I promoter region, thereby enhancing its expression. These findings provide mechanistic insights into how radiation therapy may bolster the antigen‐presenting capacity of neutrophils through IFN‐α induction, ultimately contributing to enhanced anti‐tumor immune responses.

Furthermore, we explored the therapeutic potential of adoptively transferring ex vivo‐induced ISG15^+^MHC‐I^+^ neutrophils in cancer immunotherapy [35]. A recent glioblastoma study demonstrated that engineered neutrophil‐like cells derived from human pluripotent stem cells, when combined with drug‐loaded nanomaterials, exhibit potent anti‐tumor activity, highlighting their potential for targeted immunotherapy [36]. Our study systematically defined the optimal induction conditions for generating ISG15^+^MHC‐I^+^neutrophils using radiation‐conditioned medium and IFN‐α. Although IFN‐α has inherent anti‐tumor effects, its extensive side effects have limited its widespread use in cancer therapy [37]. Here, we demonstrated that both intraperitoneal IFN‐α administration and re‐infused ISG15^+^MHC‐I^+^ neutrophils exerted anti‐tumor effects. Importantly, while reinfused neutrophils alone did not match the anti‐tumor response of PD‐1 blockade, their combination with anti‐PD‐1 therapy resulted in synergistic tumor suppression, suggesting that these neutrophils may function as immunomodulators capable of converting immunologically “cold” tumors into “hot” tumors. Further optimization—such as genetic engineering or nanomaterial‐based enhancement—may be required to maximize their therapeutic potential in future work. Additionally, increased infiltration of ISG15^+^MHC‐I^+^ neutrophils in tumor tissue correlated with improved clinical outcomes in LARC patients, suggesting their potential utility as a prognostic biomarker for immunotherapy response.

## 4 Conclusion

In conclusion, this study demonstrates that neutrophils exhibit remarkable plasticity and can acquire an immune‐activated phenotype characterized by high expression of MHC class I molecules and ISG15 in response to HFRT in LARC patients, along with potent antigen‐presenting capability. Our findings clarify neutrophil‐mediated radiotherapy‐immunotherapy synergy and propose ISG15^+^MHC‐I^+^ neutrophils as a therapeutic target for LARC (Figure 7i).

## 5 Methods

### 5.1 Human Subjects

LARC patients treated in the trials NCT04928807 (the study was registered at https://clinicaltrials.gov/) at Union Hospital, Tongji Medical College, Huazhong University of Science and Technology were involved. The patients fulfilled the following inclusion criteria: a clinical diagnosis of LARC (according to the American Joint Committee on Cancer eighth Edition criteria, histologically confirmed rectum adenocarcinoma of T3‐4/N+ by magnetic resonance imaging), an age range of 18–75 years, and with an Eastern Cooperative Oncology Group performance status of 0 or 1. Patients with a history of prior antitumor treatment were ineligible. We have collected a total of 120 blood samples from different timepoints and 78 rectal cancer tissues (28 tissues were used for scRNA‐seq and 60 tissues were used for immunofluorescence). For scRNA‐seq, LARC specimens comprised pre‐ neoadjuvant therapy (T1) and post‐neoadjuvant therapy (T3) tumor tissues from three patients (all npCR) and pre‐HFRT (T1) and post‐HFRT (T2) tumor tissues from 12 patients (10 samples from pathological complete response [pCR] patients and 12 samples from non‐pathological complete response [npCR] patients). The studies and amendments were approved by Union Hospital, Tongji Medical College, Huazhong University of Science and Technology, with Institutional Review Board (No. 0271‐89). The study complied with all ethical guidelines and obtained written informed consent from all patients.

### 5.2 Cell Lines

MC38 cells line and HL60 cell line were purchased from American Type Culture Collection (USA). All cell lines were cultured in DMEM (Gibco, USA) or RPMI1640 (Gibco, USA) supplemented with 10% fetal bovine serum (FBS) and 1% penicillin/streptomycin and maintained at 37°C in a 5% CO_2_ humidified incubator. All cells were authenticated and tested for mycoplasma contamination.

### 5.3 In Vitro Cell Proliferation Assay and CCK‐8 Assay

Cell proliferation assay was performed following the manufacturer's instructions of the Cell Counting Kit‐8 (CCK‐8). Briefly, 1 × 10^3^ cells were seeded in 96‐well plates and cultured with 100 µL medium. 20 µL of the CCK‐8 reagent (Beyotime) was added to each well one hour before the end of the incubation period, following the manufacturer's instructions. The absorbance values at 450 nm were measured using a Multimode Plate Reader (USA). CCK‐8 assay was applied to measure the half maximal inhibitory concentration (IC50) of cisplatin after treated with recombination ISG15 protein for 24 h in MC38 cells.

### 5.4 Transwell Migration Experiments

Transwell chambers (8.0 µm Pore Size, CORNING) were used to assess the cell invasion ability. Cells were seeded into the upper chamber with serum‐free 1640 medium, and medium containing 30% FBS was placed in the lower chambers and incubated for 18 h in a cell incubator. After incubation, the cells were fixed in methanol for 30 mins and then stained with Crystal Violet stain solution (Beyotime). Migration cells were evaluated under a microscope at five fields per well. All the experiments were repeated three times.

### 5.5 ROS Assay

ROS were detected by the Reactive Oxygen Species Assay Kit (Beyotime, S0033).

### 5.6 Scratch Wound Healing Assays

The MC38 cells (5×10^5^ cells per well) were seeded into 24‐well plates. When the cell confluence reaches 90%, a scratch wound was created using a sterilized pipette tip (200 µL) on confluent cells. The images of wounds were acquired with a phase‐contrast light (40×) at 0 and 24 h.

### 5.7 EdU Assay

EdU cell proliferation staining was performed using an EdU kit (BeyoClickTM, EDU‐488, China). Briefly, the MC38 cells (2 × 10^4^ cells/well) were seeded in 12‐well plates and were incubated at 37°C overnight. The MC38cells were treated with control (0.1% DMSO), NAP (0.1 µm), TMPs (1 µg/mL), or N_3_‐TMPs@NAP (NAP 0.1 µm). Subsequently, the cells were incubated with EdU for 2 h, fixed with 4% paraformaldehyde for 15 min, and then permeated with 0.3% Triton X‐100 for another 15 min. The cells were then incubated with the Click Reaction Mixture for 30 min at room temperature in a dark place, and incubated with Hoechst 33342 for 10 min.

### 5.8 Western Blot Analysis

The collected cells were lysed with RIPA buffer (#G2002, Servicebio, China), containing phosphatase inhibitors (#G2007, Servicebio, China) and 1% protease inhibitors (#G2007, Servicebio, China). Then the cell lysates were centrifuged at 12 000 rpm at 4°C for 25 min and collected the supernatants. And 5× loading buffer was added to the supernatants and boiled 10 min at 100°C water. The concentration of protein in cell lysates was detected by the enhanced BCA protein analysis kit (#G2026, Servicebio, China). The same amount of protein was loaded in each well of SDS‐PAGE gels. The protein in the gels was transferred to the PVDF membranes. Then PVDF membranes were blocked with 5% non‐fat milk at room temperature for 1 h and incubated with the primary antibody at 4°C. The next day, the membranes were washed with 1× TBST 3 times (10 min each time) and incubated with the second antibody for 1 h. Then the membranes were washed again and exposed in dark field using ECL detection reagents (#G2020, Servicebio, China). The antibodies involved in this study are detailed in Key resourses Table.

### 5.9 Quantitative Real‐Time PCR (RT‐qPCR) Assay

In brief, RNA was extracted by using TRIzol reagent (#R401‐01 RNA isolater Total RNA Extraction Reagent, vazyme, Nanjing, China). RT‐qPCR was performed by using a reverse transcription kit and PCR kit (#R323‐01 HiScript III RT SuperMix for qPCR, #Q111‐02 AceQ qPCR SYBR Green Master Mix, vazyme, Nanjing, China) referring to the manufacturer's instructions. GAPDH served as the reference gene. The primer sequences for RT‐qPCR are provided in Table S3.

### 5.10 ELISA

ELISA for IFN‐α, IFN‐β, IFN‐γ was performed according to manufacturer instructions (Human IFN gamma ELISA Kit, Human IFN alpha ELISA Kit, and Human IFN alpha ELISA Kit, Invitrogen).

### 5.11 ChIP‐qRT‐PCR

For ChIP‐qPCR, ChIP Assay Kit (#P2078, Beyotime, China) was used, following the protocol of the manufacturer's instructions. Immunoprecipitation was performed using the appropriate antibodies as follows: p65 (ab32536, Abcam, 1:50 dilution) were used for ChIP assay. Mouse IgG antibody (#61656S, Cell Signaling Technology, 1:1000 dilution) and rabbit IgG antibody (#3900S, Cell Signaling Technology, 1:1000 dilution) were used as a negative control. The primers were designed according to the promoter sequences of the genes of interest. The sequences of the ChIP‐qPCR primers are shown in Table S3.

### 5.12 Mice

The male C57BL/6J and BALB/c mice (6–7 weeks old) were purchased from WUHAN MOUBAILI BIOTECHNOLOGY Co., Ltd. All mice were experimented in strict accordance with the protocol approved by the Institutional Animal Care and Use Committee, Huazhong University of Science and Technology (No. 3633). The mice were housed at 22°C–25°C temperature, 60% ± 10% humidity, under a 12 h light/dark cycle and pathogen‐free conditions. Standard rodent laboratory diet and water were provided. Mice were randomly allocated into experimental groups.

### 5.13 Preparation of Irradiated Conditioned Medium (RTCM)

To prepare for RTCM, MC38 tumor cells were inoculated in 10 cm dishes with 10 mL of medium and replaced with fresh complete medium after 24 h. Tumor cells were irradiated with 10 Gy, and the supernatants were harvested after 48 h, filtered through a 0.22 µm filter (Corning), and stored at −80°C.

### 5.14 Tumor Models and In Vivo Treatments

MC38 (3×10^5^) tumor cells were subcutaneously injected in the right flank of C57BL/6 mice. Mouse tumor size was monitored with vernier calipers every 2–3 days. Tumor size was calculated using the formula (length×width2)/2. When the tumor volume reached approximately 100–150mm^3^, all mice were randomly divided into different groups. A dose division mode of 5Gy×3 was used for local radiotherapy of mouse tumors. The anti‐mouse programmed cell death protein‐1 (αPD‐1) was administered intraperitoneally (200 µg/per mouse; RMP1‐14, BioXCell, West Lebanon, NH) every 3 days for a total of three times. As for the Ly6G antibody treatment group, mice were injected with 50 µg/mouse of anti‐Ly6G Ab (BE0075, BioXCell). As for the neutrophil adoptive delivering group, we split neutrophils from the bone marrow of 5‐week‐old male C57 mice, stimulated the neutrophils with IFN‐α (10^4^U/mL, MCE) or RTCM (4 Gy) for 12 h, and performed the transfusion of the treated neutrophils through the tail vein (5 × 10^6^) [38].  For IFN‐α treatment, recombinant IFN‐α protein (25U/g, MEC) was intraperitoneally injected three times a week for a total of two weeks. As for the T cell depletion group, mice were injected with 200 µg/mouse of anti‐mouse CD8α Ab (BE0061, BioXCell). As for radiotherapy, animals were exposed to the indicated dose of radiation by 6MV X rays at 600MU/min (Varian, USA). For survival studies, mice were euthanized when the tumor volume reached 1500 mm^3^ or ulcer ≥ 5 mm.

### 5.15 Bulk RNA‐Seq Analysis

Mouse primary neutrophils samples were lysed with TRIzol reagent (Invitrogen, CA, USA) and sent for transcriptome sequencing in liquid nitrogen (BGI, Shenzhen, China). Total RNA was extracted from mouse primary neutrophils. RNA quantification and integrity were measured by an Agilent 2100 Bioanalyzer (Agilent RNA 6000 Nano Kit). The mRNA was purified using oligo (dT) ‐attached magnetic beads and DNA probes to eliminate contamination from other nucleic acids. The mRNA was then fragmented and reverse transcribed into cDNA for the construction of the final library. The RNA‐seq libraries were prepared for sequencing at BGI Genomics (Shenzhen, China). For RNA‐seq analysis, the raw sequencing data were filtered with SOAPnuke (v1.4.0) to obtain clean data in FASTQ format and then mapped to the reference genome using HISAT (v2.1.0). The clean data were mapped to the assembled unique gene by Bowtie2 (v2.2.5), and the expression level of genes was calculated by RSEM (v1.2.8). Differential expression analysis was performed using DESeq with a Q value corrected (Q value < 0.05) by Bonferroni correction. The Dr.Tom online platform of BGI was used for data analysis (https://biosys.bgi.com). Analysis was performed for differentially expressed genes (Q < 0.05 and fold change ≥1.41) by Ingenuity Pathway Analysis (IPA, Genechem, Shanghai). The volcano plot was generated using ‘ggplot2 (v3.4.4)’ package in R (Q < 0.05 and fold change ≥1.41).

### 5.16 Single Cell RNA‐Seq (scRNA) Analysis

All sample collection procedures were in accordance with clinical routine. Single‐cell suspensions were generated using the Tumor Dissociation Kit (Miltenyi Biotec) according to the manufacturer's protocol. A Dead Cell Removal Kit (Miltenyi Biotec) was applied to eliminate dead cells from the single‐cell suspensions. CD45^+^ cells were sorted from processed LARC tumors. Cell viability in single‐cell suspensions was >90%. The concentration of single‐cell suspension was adjusted to 700‐1200 cells/uL. The cell suspension was loaded into Chromium microfluidic chips to establish single‐cell gel beads in emulsion (GEMs) for the directed retrieval of approximately 5000 cells and barcoded with the Chromium Controller (10x Genomics). RNA from the barcoded cells was subsequently reverse transcribed, and sequencing libraries were constructed with reagents from a Chromium Single Cell 3’ Reagent Kit v3 (10x Genomics) according to the manufacturer's instructions. Sequencing was performed with the Illumina sequencing platform (NovaSeq) in Novogene.

Raw reads were demultiplexed and mapped to the reference genome by 10x Genomics Cell Ranger pipeline (https://support.10x genomics.com/ single‐cell‐ geneexpression/software/pipelines/latest/what‐is‐cell‐ranger) using default parameters. For each gene and each cell barcode filtered by Cell Ranger, unique molecule identifiers (UMIs) were counted to construct digital expression matrices. The resulting analysis files for each sample were aggregated using the cellranger aggr pipeline, which performed a between‐sample normalization step and merged two samples into one. The Seurat package was used to normalise data, dimensionality reduction, clustering, and differential expression. A gene with expression in more than three cells was considered to be expressed, and each cell was required to have at least 200 expressed genes. Raw UMI counts were normalized, and most variable genes were detected by the FindVariableFeatures function. Principal components analysis (PCA) was performed using variable genes. For clustering, we used the function FindClusters, which implements shared nearest neighbor based on PCA using the first 20 principal components with a resolution of 0.6.

Differential abundance analysis of clusters in different conditions (T1 vs. T3) was performed by using miloR (version 2.0.0). The clusterProfiler R package and the org.Hs.e.g.,.db R package was applied to perform Gene Ontology (GO), and Kyoto Encyclopedia of Genes and Genomes (KEGG). R package Monocle2 (version 2.32.0), Monocle3 (version 1.3.1), Slingshot (version 2.12.0), and Cytotrace2 (version 1.0.0) were used to estimate the developmental pseudotime of neutrophils. To identify potential cell‐cell interactions, the R package CellChat (version 1.6.1) was used to evaluate the expression of pairs of ligands and receptors within cell populations from the Seurat object. The number of interactions among cell types was visualized as heatmap to show the aggregated cell‐cell communication network and signaling. All visualizations were done using three R packages of ggplot2, scRNAtoolVis, and ggrepel.

### 5.17 Primary CD8^+^ T Cell Preparation

Spleens of C57BL/6 WT mice (male, 6–8 weeks old) were harvested and filtered through a 40 µm cell strainer to produce a single‐cell suspension. After red blood cell lysis, the single‐cell suspension was purified using the MojoSort Mouse CD8^+^ T Cell Isolation Kit (BioLegend, USA) according to the manufacturer's instructions to obtain CD8^+^ T cells. The purity of CD8^+^ T cells isolation were assayed by flow cytometry. Purified CD8^+^ T cells were seeded in the anti‐CD3‐coated (5ug/mL, BioLegend, clone 145‐2C11) 24 well‐plates in complete RPMI medium (10% FBS, 1% Pencillin/Streptomycin, 1 mM Sodium Pyruvate, 10 mm HEPES, 50 µm β‐mercaptoethanol, 20 ng/mL interleukin (IL)‐2, and 5 ug/mL anti‐mouse CD28 antibodies (BioLegend, clone 37.51) and cultured for 72 h for CD8^+^ T cell activation.

### 5.18 Primary Neutrophil Preparation

Bone marrow of C57BL/6 WT mice (male, 6–8 weeks old) was harvested and filtered through a 40 µm cell strainer to produce a single‐cell suspension. After red blood cell lysis, the single‐cell suspension was purified using the MojoSort Mouse Neutrophils Isolation Kit (BioLegend, USA) according to the manufacturer's instructions to obtain Neutrophils. The purity of Neutrophils isolation was assayed by flow cytometry. Cells (5 ×104) were added to 96‐well cell culture plates in a total volume of 200 µL of culture medium. To maintain neutrophil activity, we added Lipopolysaccharides (LPS, MCE, HY‐D1056) at a concentration of 100 ng/mL.

### 5.19 Neutrophil and T Cell Co‐Culture

For proliferation assay, activated CD8^+^ T cells were labeled with 3 µm carboxyfluorescein diacetate succinimide ester (CFSE, BioLegend) at 37°C for 8 min and then co‐cultured with pretreated neutrophils in RPMI‐1640 medium supplemented with 10% fetal bovine serum, 20 ng/mL interleukin (IL)‐2 (BioLegend, USA), and 50 µm β‐mercaptoethanol. Neutrophil were seeded with activated T cells at a ratio of 1:10. After co‐culture for another 72 h, the proliferation of CD8^+^ T cells was analyzed using flow cytometry.

### 5.20 Flow Cytometry

The flow cytometry samples involved in this study included tumor tissues from mice, as well as neutrophils and T cells applied in in vitro functional experiments. Tumor tissues were cut into small pieces and digested in RPMI 1640 medium (without FBS) containing 0.32 mg/mL collagenase V, 0.5 mg/mL hyaluronidase, and 5 µg/mL DNase I for 1 h at 37°C and filtered through a 70 um filter to generate a single‐cell suspension. Cells were pre‐incubated with TruStain FcX (anti‐mouse CD16/32) antibody (clone 93, BioLegend) and stained with a Zombie NIR Fixable Viability Kit (BioLegend) for 20 min on ice followed by immunostaining. Appropriate cell surface marker antibodies were selected and stained in the dark at 4°C for 30 min, and/or fixed (1% polyformaldehyde, Biosharp)/permeated (Intracellular Staining Perm Wash Buffer, Biolegend), and intracellular antibodies (intracellular markers IFN‐γ, Biolegend) were stained at 4°C for 30 min. In addition, for intracellular cytokine staining (IFN‐γ), cells were stimulated ex vivo with 100 ng/mL PMA, 1ug/mL ionomycin, and 1.5ug/mL monenamycin for 4–6 h at 37°Cin 10% FBS and 1% penicillin/streptomycin. All samples were detected by a CytoFLEX flow cytometer (Beckman Kurt Trading Co, Ltd, USA), and all data were analyzed in FlowJo software version 10.8.1. The flow cytometry antibodies involved in this study are detailed in the Key resourse Table. Gating strategies are displayed in Figure S10.

### 5.21 Cell Deconvolution of the Human Pan‐Cancer Bulk RNA‐Seq Datasets

Our LARC scRNA‐seq dataset with annotated mono‐macrophages signature was used as a single‐cell reference matrix. We computed the average expression matrices for each mono‐macrophages clusterusing the AverageExpression function in the Seurat object. The single‐cell reference matrix, average expression matrices, and bulk RNA‐seq data of pan‐cancer in TCGA were utilized as inputs, while the other parameters were set as the algorithm's default options.

### 5.22 Statistical Analyses

To estimate the statistical significance of differences between two groups, we used a paired or un‐paired Student's *t*‐tests to calculate two‐tailed P values. Mann‐Whitney test was used for the comparison of nonparametric data between two groups. One‐way analysis of variance (ANOVA) with Tukey's post hoc comparisons was performed when more than two groups were compared. Survival analysis was performed using Kaplan‐Meier curves and evaluated with logrank Mantel‐Cox tests. Error bars indicate the standard error of the mean (SEM) unless otherwise noted. *p* values are labeled in the figures. *P* values were denoted as follows: * *p* < 0.05, ** *p* < 0.01, *** *p* < 0.001, **** *p* < 0.0001. Apart from scRNA‐seq analysis, statistical analyses were performed by using GraphPad Prism (version 8.0).

## Author Contributions

Lichao Liu, Haihong Wang, Mingjie Li contributed equally to this work. Lichao Liu: validation, investigation, methodology, writing – original draft. Haihong Wang: investigation and methodology. Mingjie Li: investigation and methodology. Qian Xu: investigation and methodology. Linlin Zheng: formal analysis, writing – review and editing. Xiaorong Dong: formal analysis, methodology, writing – review and editing. Jinghua Ren: formal analysis, methodology, writing – review and editing. Chaoqun Han: formal analysis, writing – review and editing. Peng Zhang: formal analysis and methodology. Kaixiong Tao: methodology, writing – review. Zhenyu Lin: conceptualization, resources, funding acquisition, validation and investigation. Tao Zhang: conceptualization, resources, funding acquisition, validation, investigation. All authors have read and approved the article.

This work was supported by the National Natural Science Foundation of China (No. 82573302 and No. 82203808), Chinese Society of Clinical Oncology (CSCO)‐Tongshu Oncology Research Fund (Y‐tongshu2021/qn‐0205), CSCO Research Fund (Y‐2022HER2AZZD‐0374), National Key Technology Research and Development Programme of Hubei Province (No. SHFZ202400031), Scientific Research Fund of Wuhan Union Hospital‐Joint Research Fund (2023XHYN003), Natural Science Foundation of Hubei Province of China‐Hengrui Medicine Joint Fund for Innovative Development (2025AFD771), Shenzhen Basic Research Fund (JCYJ20240813153408011), “Jiebang Guashuai” Project for Clinical Specialties of Wuhan Union Hospital (2025JBGS1003), Huazhong University of Science and Technology Interdisciplinary Research Support Program (2025JCYJ065), and Open Research Fund of Hubei Province Key Laboratory of Precision Radiation Oncology (No. 2024ZLJZFL012).

## Ethics Statement

All mice were experimented on strict accordance with the protocol approved by the Institutional Animal Care and Use Committee, Huazhong University of Science and Technology (No. 3633). The human samples were obtained from patients treated in the trials NCT04928807. The studies and amendments were approved by Union Hospital, Tongji Medical College, Huazhong University of Science and Technology, with Institutional Review Board (No. 0271‐89).

## Conflicts of Interest

The authors declare no conflicts of interest.

## Supporting information




**Supporting File**: advs74766‐sup‐0001‐SuppMat.docx.

## Data Availability

The sequencing data (including single‐cell RNA‐seq and bulk RNA‐seq) reported in this paper are deposited in the NCBI Gene Expression Omnibus (GEO) database with accession numbers: GSE278406 and GSE316027 (URL: https://www.ncbi.nlm.nih.gov/geo/). Accession numbers are also listed in the key resources table.
