# Supplementary material for: Hypofractionated Radiotherapy Induces ISG15+MHC‐I+ Neutrophils to Augment Anti‐Tumor Immunity and Prime Immune Checkpoint Blockade Responses in Rectal Cancer
Source: Adv Sci (Weinh). 2026 Mar 13;13(29):e17450. doi: 10.1002/advs.202517450 (PMC13205831; doi:10.1002/advs.202517450)
Supplement: Supplementary file 1 — Supporting File: advs74766‐sup‐0001‐SuppMat.docx. [file ADVS-13-e17450-s001.docx]

**
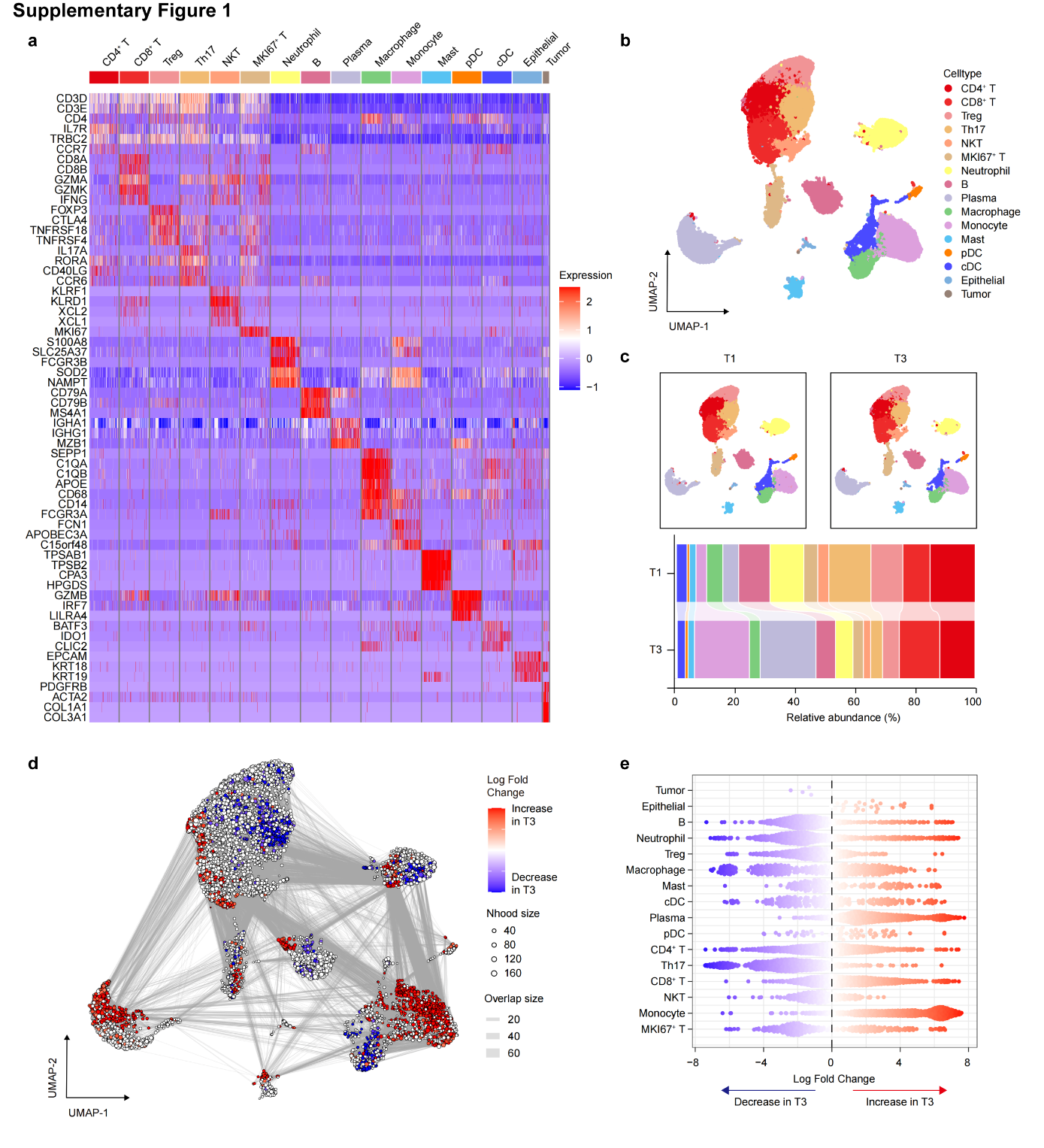
Supplementary Material and Methods**

**Supplementary Figure 1**

a. Heatmap showing markers of different cell populations.

b. UMAP showing different immune cell populations which have been identified.

c. UMAP showing classification of all clusters in each condition (T1 vs. T3). Boxplots showing the alterations of cell clusters between T1 and T3. Color of each cluster refers to (b). T1: pre-neoadjuvant therapy. T3: post-neoadjuvant therapy.

d. Neighborhood graph of all cells based on Milo differential abundance testing. Blue and red colours indicate the log2-fold difference between T1 and T3 conditions. Neighborhoods that increased in T3 are shown in red. Neighborhoods decreased in T3 are shown in blue. The point size represents the number of cells in a neighbourhood, and the edge thickness indicates the number of cells shared between pairs of neighbourhoodse. Beeswarm plot showing the distribution of log2-fold differences in neighborhoods in different clusters. Colors and descriptions are represented similarly to (d).

**
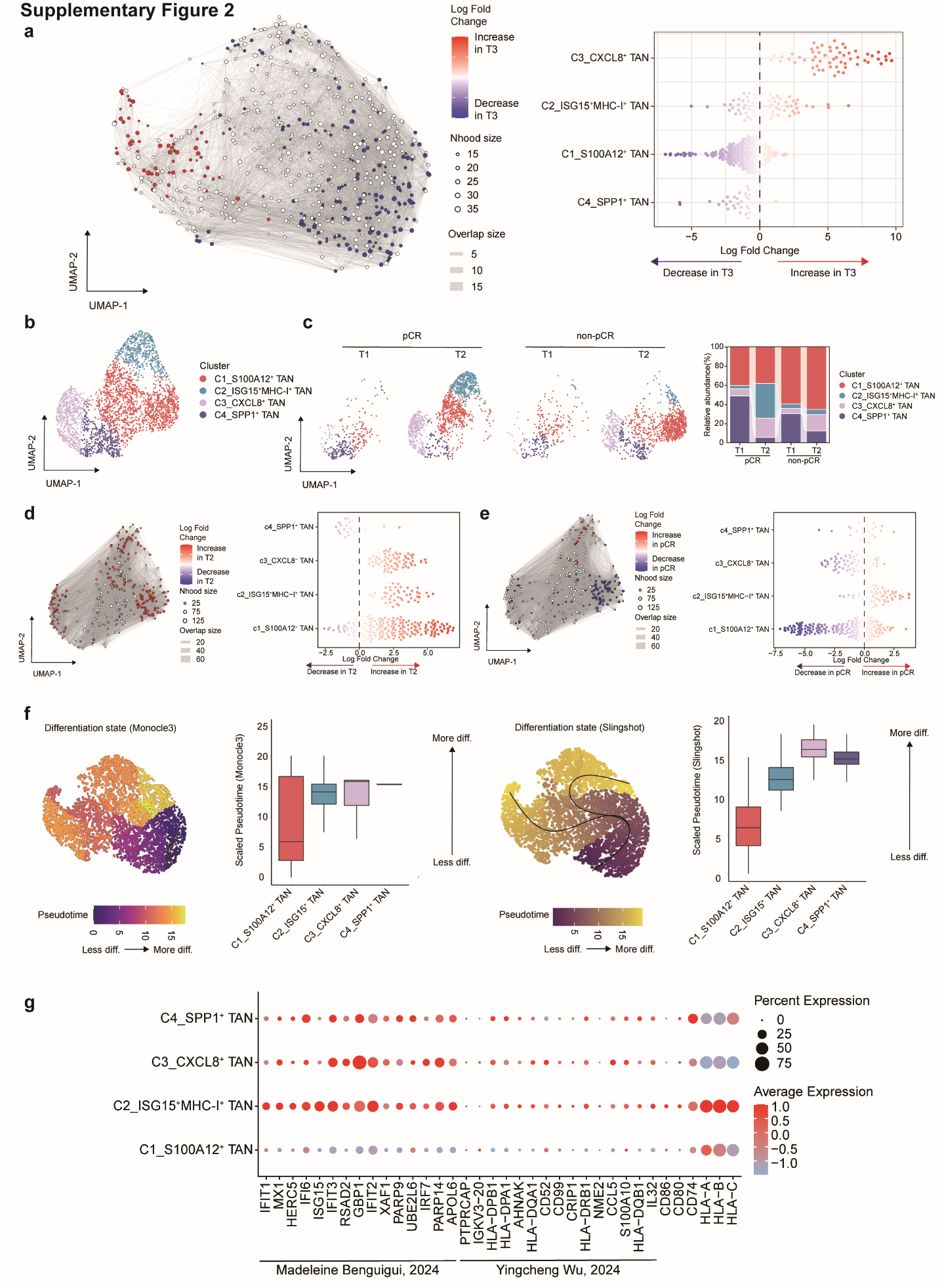
**

**Supplementary Figure 2**

a. Neighborhood graph of neutrophils using Milo differential abundance testing. The analysis was performed similarly to Figure S1d. Beeswarm plot showing the distribution of log2-fold differences in neighborhoods in different clusters.

b. UMAP showing different neutrophils populations which have been identified.

c. UMAP showing classification of neutrophils clusters in each condition (T1 vs. T2). Boxplots showing the alterations of cell clusters between T1 and T3. T1: pre-HFRT. T2: post-HFRT.

c. Differentiation state estimated by Monocle3 (leftt), and box plots showing the Pseudotime of different neutrophil clusters (right).

d. Neighborhood graph of neutrophils using Milo differential abundance testing. The analysis was performed similarly to a. Blue and red colours indicate the log2-fold difference between T1 and T3 conditions. Neighborhoods that increased in T2 are shown in red. Neighborhoods decreased in T2 are shown in blue.

e. Neighborhood graph of neutrophils using Milo differential abundance testing. The analysis was performed similarly to a. Blue and red colours indicate the log2-fold difference between pCR and npCR conditions. Neighborhoods that increased in pCR are shown in red. Neighborhoods decreased in npCR are shown in blue.

f. Differentiation state estimated by Monocle3 and Slingshot, and box plots showing the Pseu dotime of different neutrophil clusters. The black lines represent the developmen tal pathway estimated by Slingshot.

g. Comparison of characteristic Genes between neutrophil subsets in this study with published mouse neutrophil states

**
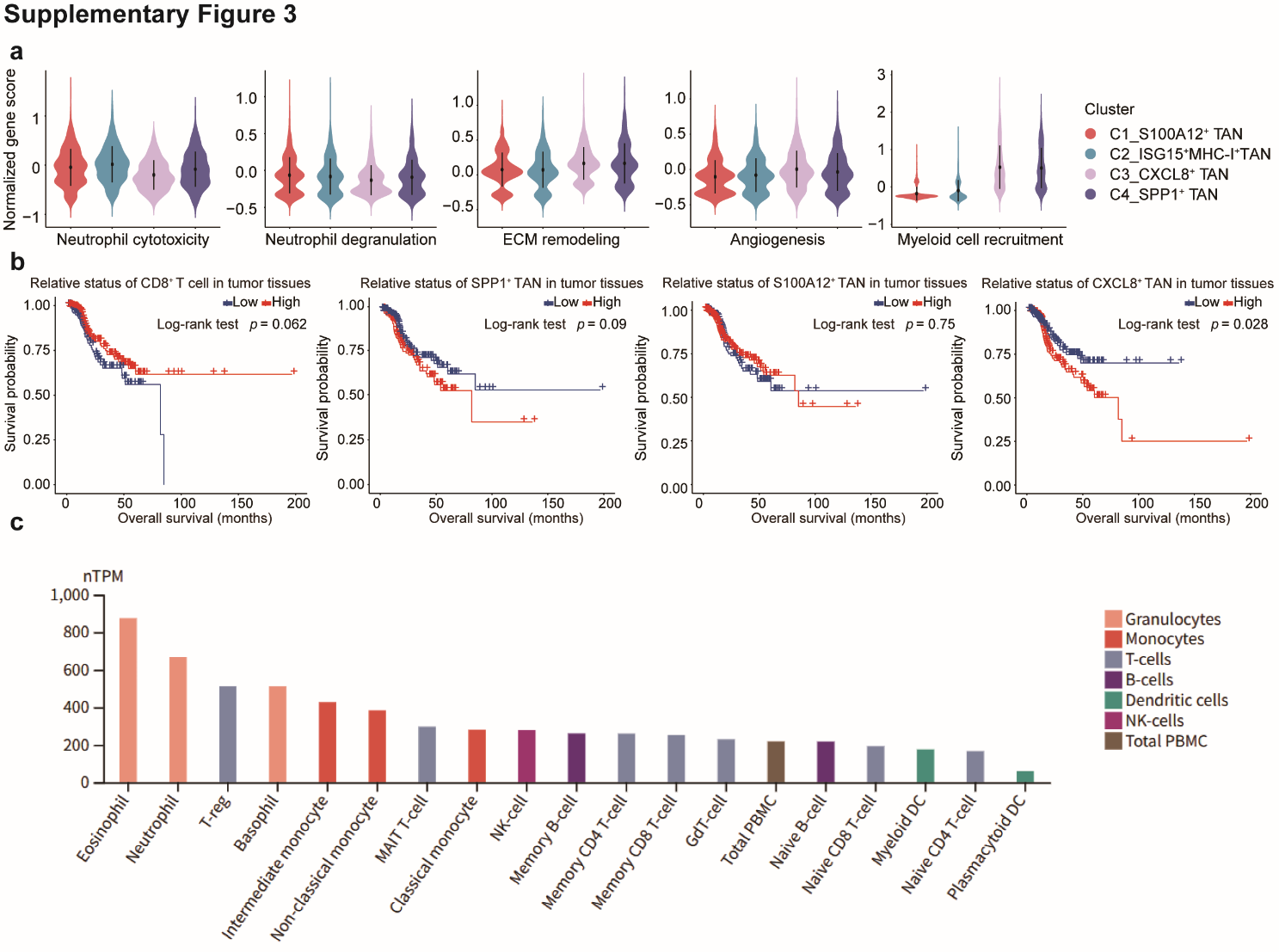
**

**Supplementary Figure 3**

a. Expression of functional gene sets in neutrophil clusters before and after SIC by using Add Module Score function of Seurat package.

b. Kaplan–Meier curves for overall survival according to the relative status of CD8+, SPP1+, S100A12+ and CXCL8+ in tumor tissues after radiation therapy in TCGA database. Statistical analysis was performed using two-sided log rank test.

c. The expression of ISG15 resulting from the internal normalization pipeline for 18 immune cell types and total peripheral blood mononuclear cells (PBMC). The resulting transcript expression values calculated as nTPM.

**
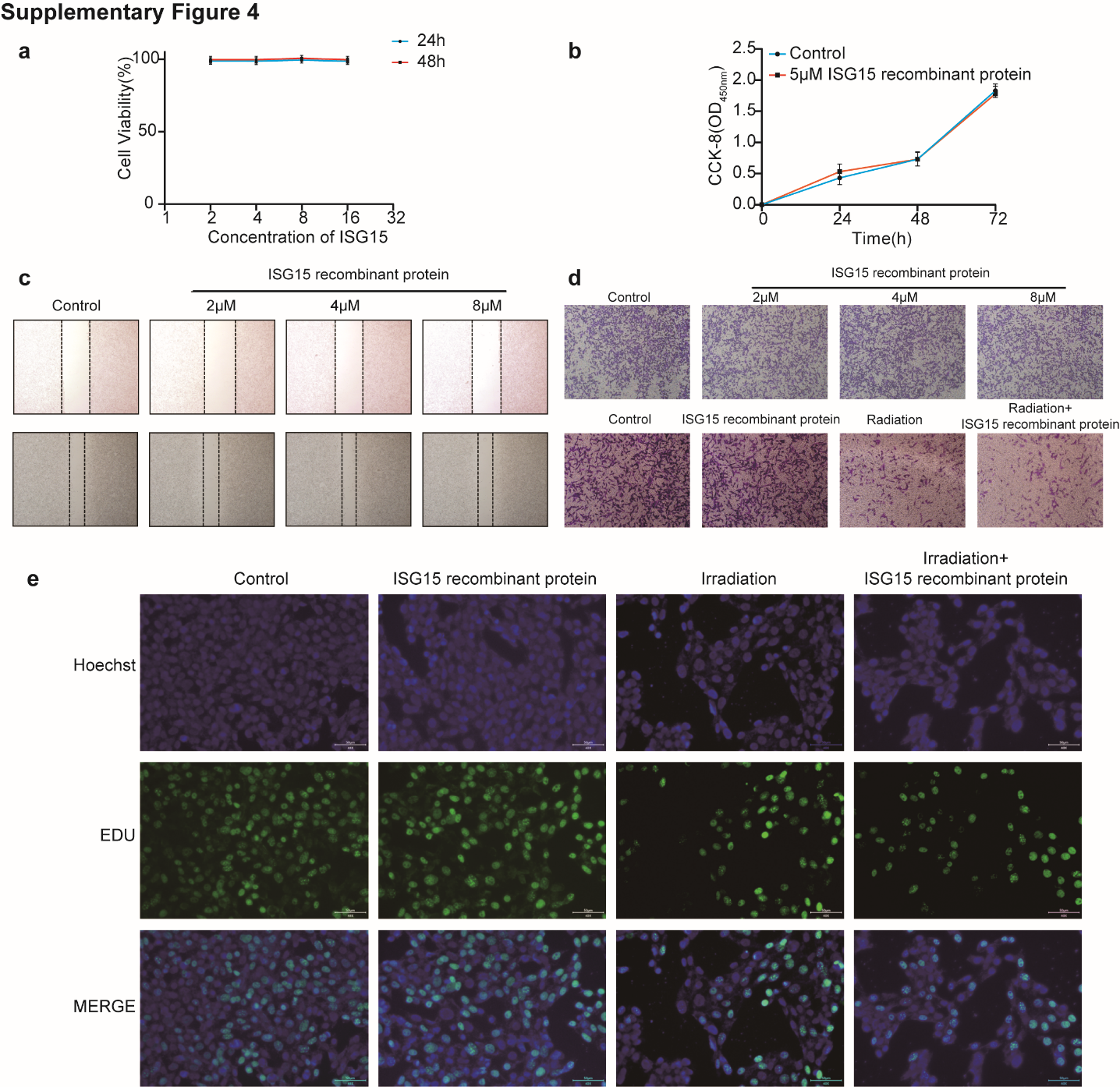
**

**Supplementary Figure 4**

a. Cell viability percentage of MC38 cells treated with different concentrations of recombinant ISG15 protein for 24 hours or 48 hours.

b. CCK-8 assay for MC38 cells treated with either the control or 5μM ISG15 recombinant protein for 24h, 48h and 72h.

c. Representative images of the wound healing assay for MC38 cells treated with 2μM, 4μM and 8μM ISG15 recombinant protein for 24h.

d. Representative images of transwell assay for MC38 cells treated with 2μM, 4μM and 8μM ISG55 recombinant protein for 24h.

e. Representative images of EDU assay for MC38 cells treated with 5μM ISG15 recombinant protein and irradiation for 24h.

**
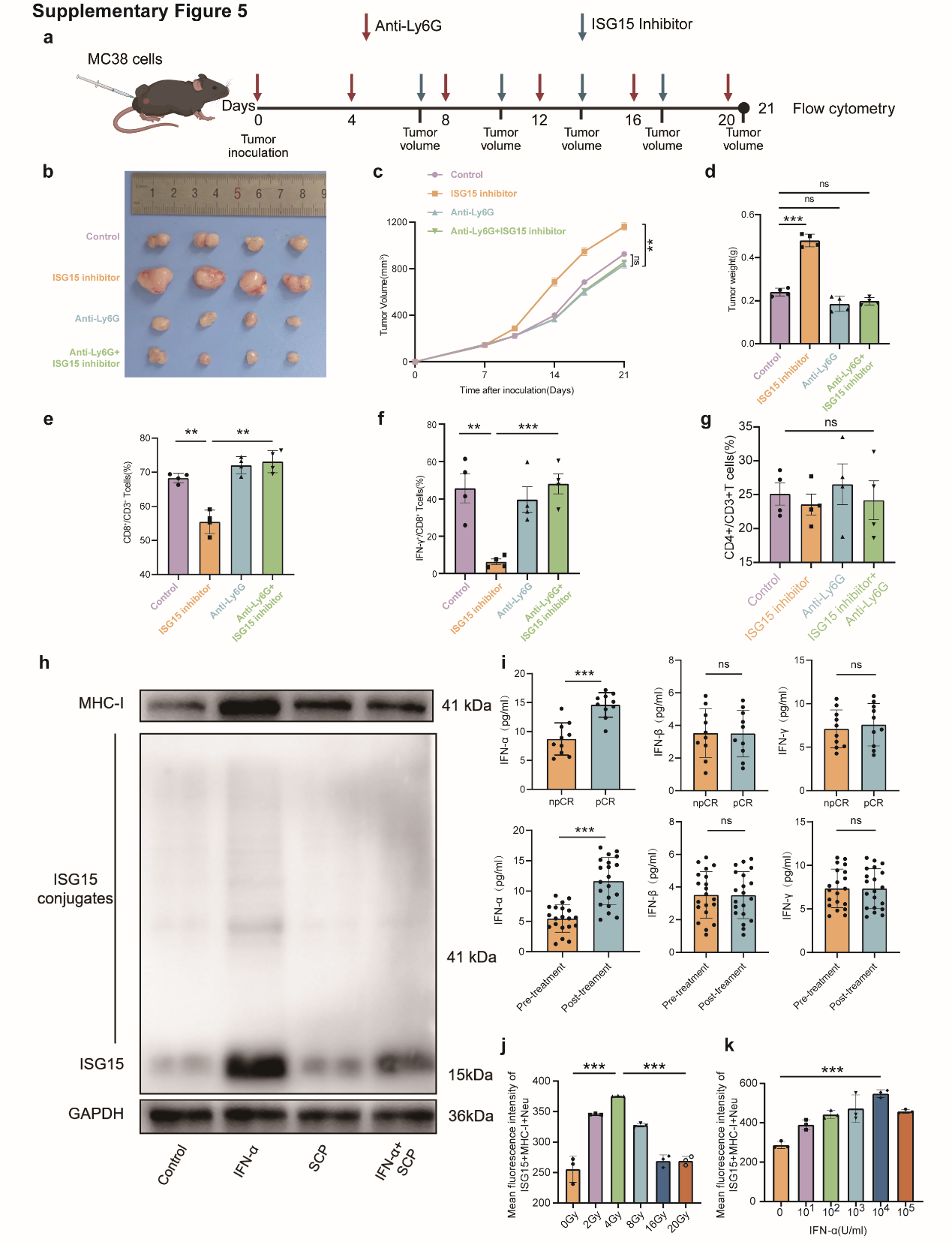
**

**Supplementary Figure 5**

a. Schematic diagram of MC38 subcutaneous tumor mouse model. C57BL/6 mice were subcutaneously inoculated with MC38 cell line (1×10⁶ cells/mouse) in the right lateral thigh. When tumors reached 100-150 mm³, mice were randomly assigned to four groups: Control, anti-Ly6G, ISG15 inhibitor and Combination group. Tumor growth was monitored.

b. Representative images of tumors in mice treated as described above.

c. Tumor growth of MC38 tumor-bearing C57BL/6 mice treated with the indicated treatments (n=4 per group).

d. Tumor weights in mice treated as described above (n=4 per group). One-way ANOVA.

e. Percentages of CD8^+^ T cells (CD8^+^/CD3^+^ T cells) in tumor tissue (n=3 per group).

f. Percentages of IFN-γ^+^CD8^+^ T cells (IFN-γ^+^/CD8^+^ T cells) in tumor tissue (n=3 per group). One-way ANOVA.

g. Percentages of CD4^+^ T cells with different treatment assessed by flow cytometry (n=3 per group). One-way ANOVA.

h. Western blot of ISG15 and MHC-I in primary neutrophils stimulated with or without SCP and IFN-α.

i. Human serum cytokine concentrations of IFN-α, IFN-β, and IFN-γ detected using ELISA. Two-sided unpaired t test.

j. Percentages of ISG15^+^MHC-I^+^ neutrophils after different irradiation dose assessed by flow cytometry (n=3 per group). One-way ANOVA.

k. Percentages of ISG15^+^MHC-I^+^ neutrophils after different concentrations of IFN-α assessed by flow cytometry (n=3 per group). One-way ANOVA.

∗*p* < 0.05; ∗∗*p* < 0.01; ∗∗∗*p* < 0.001; ∗∗∗∗*p* < 0.0001; ns, not significant.


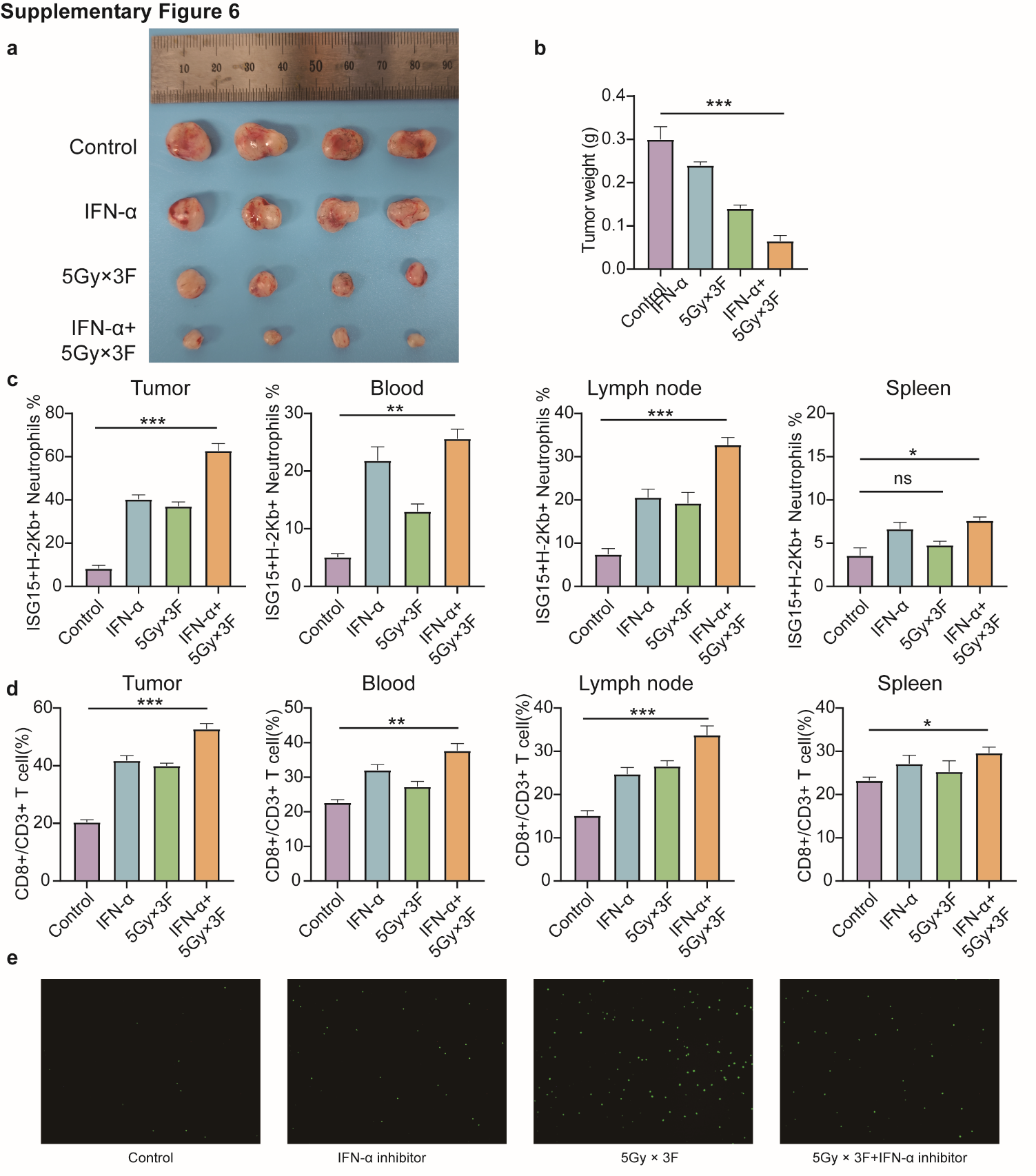


**Supplementary Figure 6**

a. Representative images of tumors in mice. C57BL/6 mice were subcutaneously inoculated with MC38 cell line (1×10⁶ cells/mouse) in the right lateral thigh. When tumors reached 100-150 mm³, mice were randomly assigned to four groups: Control, IFN-α, 5Gy×3F and Combination group. Tumor growth was monitored.

c. Tumor growth of MC38 tumor-bearing C57BL/6 mice treated with the indicated treatments (n=4 per group).

e. Percentages of ISG15^+^H-2Kb^+^ neutrophils in tumor, Blood, lymph node, and spleen assessed by flow cytometry. (n=4 per group). One-way ANOVA.

d. Percentages of CD8^+^ T cell in tumor, blood, lymph node assessed, and spleen by flow cytometry (n=4 per group). One-way ANOVA.

e. ROS staining of primary neutrophils with different treatments for 24h.

∗*p* < 0.05; ∗∗*p* < 0.01; ∗∗∗*p* < 0.001; ∗∗∗∗*p* < 0.0001; ns, not significant.


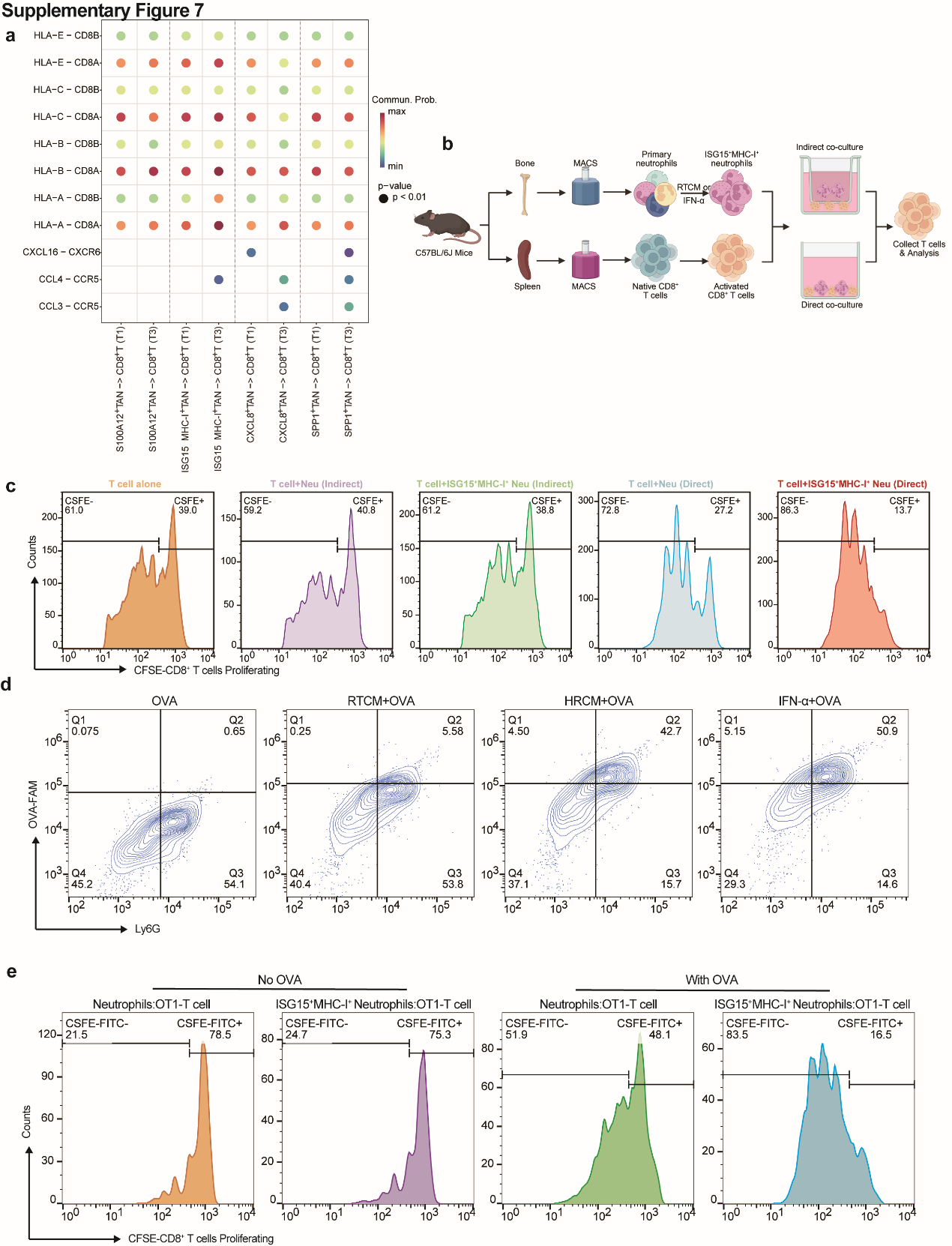


**Supplementary Figure 7**

a. The ligand–receptor pairs exhibit significant changes in specificity between any one of the neutrophil clusters and CD8^+^ T cluster in T1 versus T3. The panel shows that CD8^+^ T expresses receptors and receives ligand signals from other neutrophils.

b. Schematic diagram of co-culture of neutrophils and CD8+T cells

c. Direct or indirect co-culture systems of neutrophils and naive T cells were established in the presence of anti-CD3/CD28 antibodies. Representative flow histograms of proliferating CD8^+^ T cells.

d. Representative flow cytometry contour plots of OVA-FAM⁺Ly6G⁺ neutrophils.

e. With or without OVA co-culture systems of neutrophils and OT1 T cells. Representative flow histograms of proliferating CD8^+^ T cells.


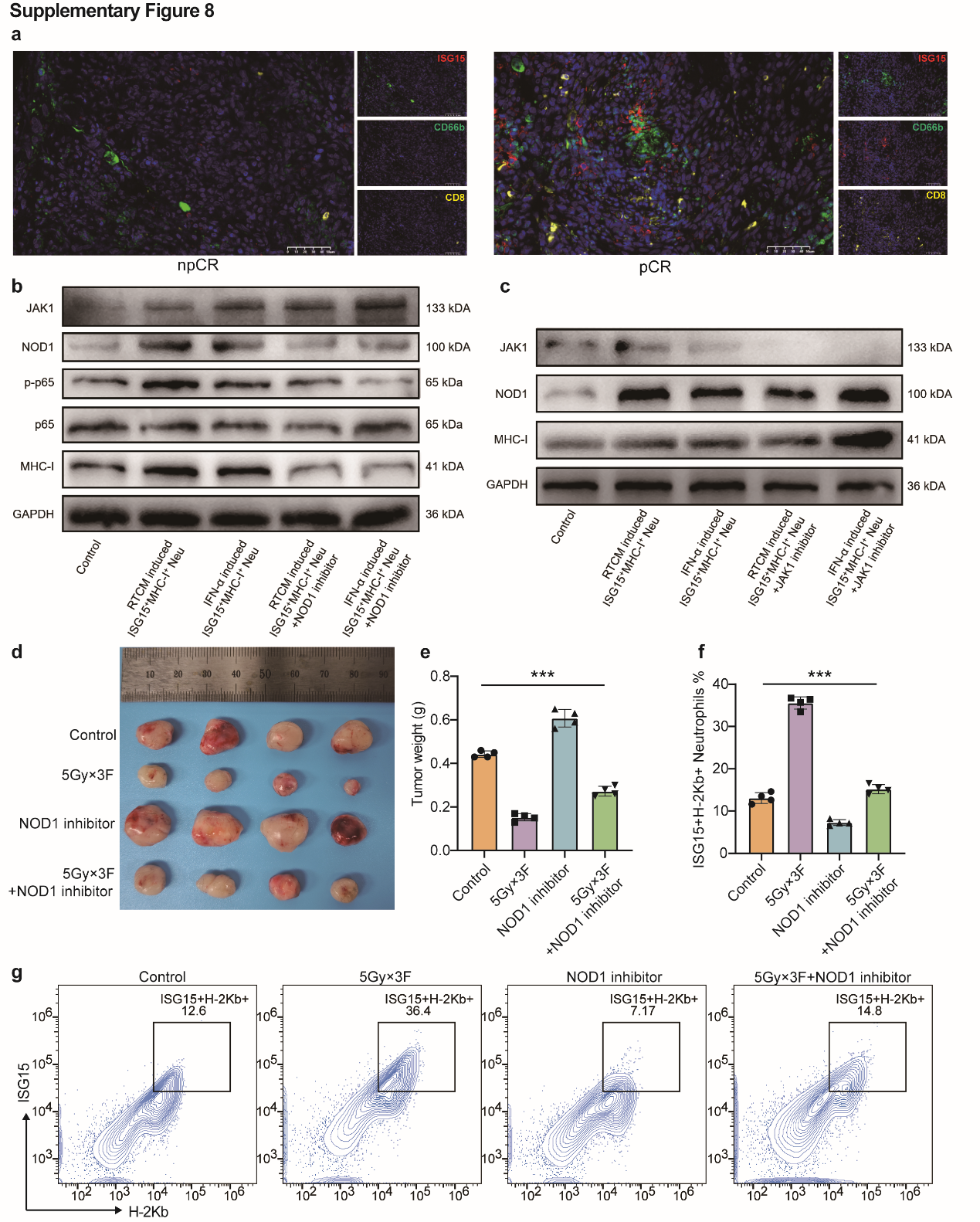


**Supplementary Figure 8**

a. Association between ISG15^+^CD66b^+^ neutrophils and CD8^+^ T cells using mIHC in the post-neoadjuvant therapy tumor sample of LARC patient. Scale bars, 50 μm.

b. Western blotting for the expression of Jak1, MHC class I, NOD1, p-p65 and p65 in the primary neutrophil, ±NOD1-IN-1.

c. Western blotting for the expression of Jak1, MHC class I, NOD1, p-p65 and p65 in the primary neutrophil, ±JAK1-IN-13.

d. C57BL/6 mice were subcutaneously inoculated with MC38 cell line (1×10⁶ cells/mouse) in the right lateral thigh. When tumors reached 100-150 mm³, mice were randomly assigned to four groups: Control, 5Gy×3F, NOD1-IN-1 and Combination group. Tumor growth was monitored. Representative images of tumors in mice treated as described above (n=6-8 per group).

e. Tumor weights in mice treated as described above (n=4 per group). One-way ANOVA.

f. Percentages of ISG15^+^H-2Kb^+^ neutrophils in tumor assessed by flow cytometry (n=3 per group). One-way ANOVA.

g. Representative flow cytometry contour plots of ISG15^+^H-2Kb^+^ neutrophils.


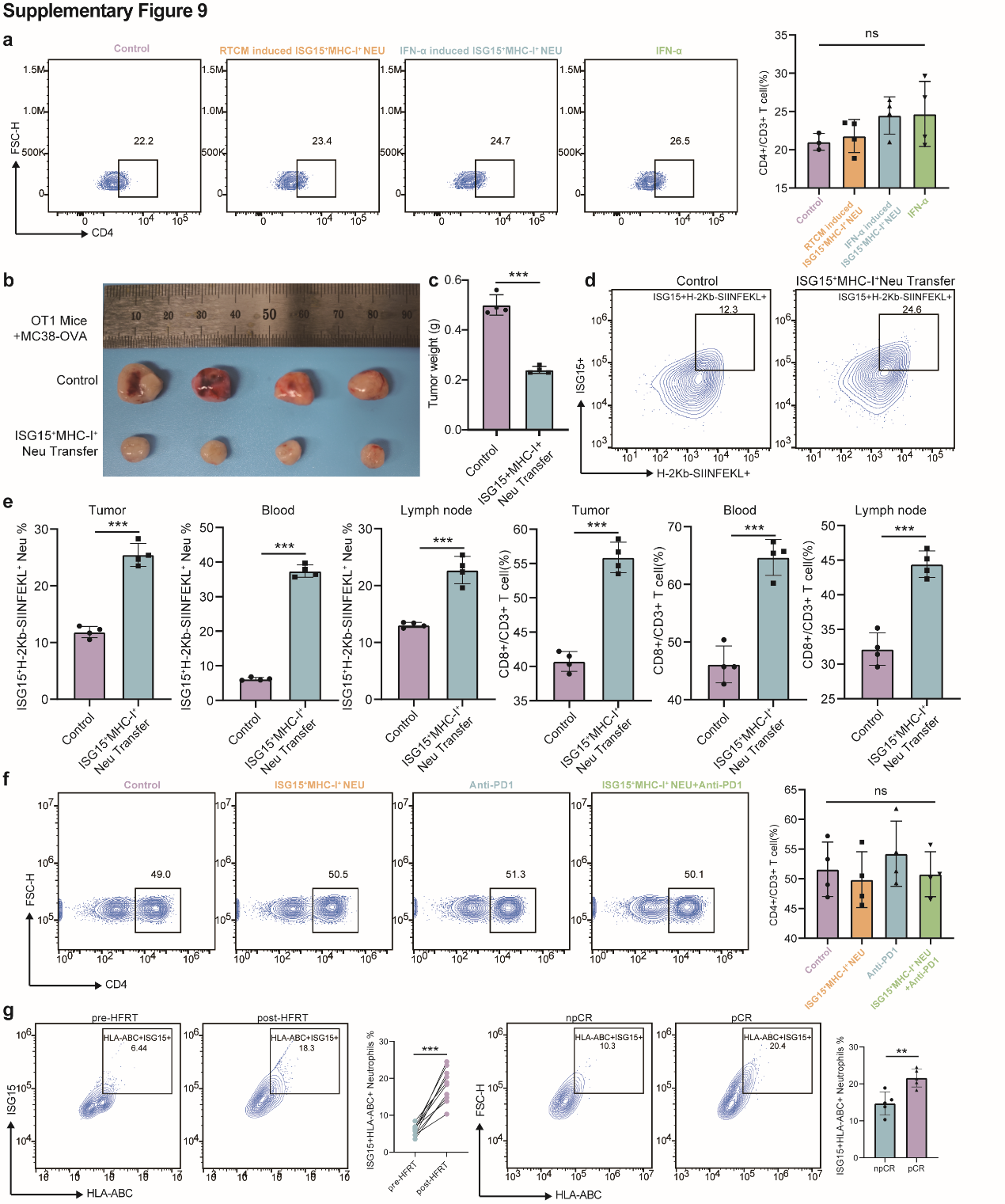


**Supplementary Figure 9**

a. Representative flow histograms (Left) and percentages (Right) of CD4^+^ T cells with different treatment assessed by flow cytometry. (n=3 per group)

b. OT1 mice were subcutaneously inoculated with MC38-OVA cell line (1×10⁶ cells/mouse) in the right lateral thigh. When tumors reached 100-150 mm³, mice were randomly assigned to four groups: Control, ISG15^+^MHC-I^+^ neutrophil reinfusion (n=6-8 per group). Representative images of tumors in mice treated as described above.

d. Representative flow cytometry contour plots of ISG15^+^H-2Kb^+^ neutrophils in tumor.

e. Percentages of ISG15^+^H-2Kb^+^ neutrophils in tumor, Blood, lymph node assessed by flow cytometry. Percentages of CD8^+^ T cell in tumor, Blood, lymph node assessed by flow cytometry (n=3 per group). One-way ANOVA.

f. Representative flow histograms (Left) and percentages (Right) of CD4^+^ T cells with different treatment assessed by flow cytometry. (n=3 per group)

g. Representative flow cytometry contour plots and corresponding statistical graphs of the proportion of ISG15⁺HLA-ABC⁺ neutrophils in human peripheral blood (pre-HFRT=10, post-HFT=10, npCR=5, and pCR=5). Two-sided unpaired t test (Left). Two-sided paired t test (Right).

∗*p* < 0.05; ∗∗*p* < 0.01; ∗∗∗*p* < 0.001; ∗∗∗∗*p* < 0.0001; ns, not significant.

**
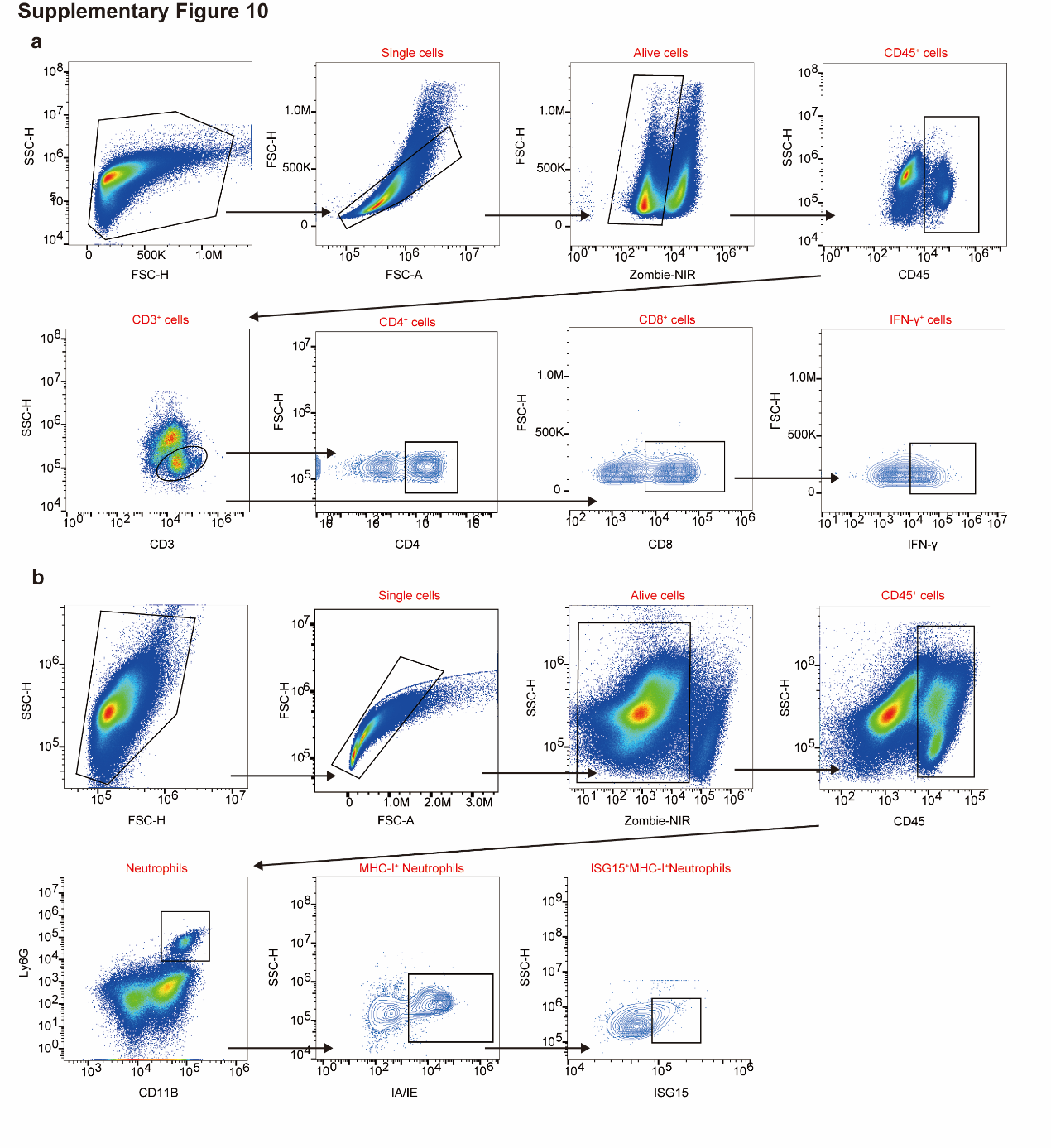
**

**Supplementary Figure 10 Gating strategy for flow cytometry in this study.**

a. Gating strategy for flow cytometry of T cells in tumor.

B. Gating strategy for flow cytometry of neutrophils in tumor.

**TableS1.Clinical characteristics of patients involved in the predict cohort**

| **Patient ID** | **Ages (Years)** | **Sex** | **Tumor location** | **Length of tumor lesion (cm)** | **Clinical TNM stage** | **EMVI** | **MRI-CRM** | **Treatment** | **Pathological remission** | **MMR status** | **mrTRG** |
| --- | --- | --- | --- | --- | --- | --- | --- | --- | --- | --- | --- |
| 1 | 65 | male | lower | 8.20 | IIIB(T3N1M0) | negative | negative | HFRT+NIC | pCR | pMMR | 2 |
| 2 | 58 | female | middle | 3.60 | IIIB(T3N2M0) | positive | positive | HFRT+NIC | non-pCR | pMMR | 4 |
| 3 | 72 | male | lower | 5.00 | IIA(T3N0M0) | negative | positive | HFRT+NIC | non-pCR | pMMR | 3 |
| 4 | 60 | male | middle | 2.80 | IIB(T4aN0M0) | negative | negative | HFRT+NIC | pCR | pMMR | 3 |
| 5 | 53 | female | lower | 4.80 | IIA(T3N0M0) | negative | positive | HFRT+NIC | pCR | pMMR | 3 |
| 6 | 60 | male | middle | 3.20 | IIIB(T3N2aM0) | positive | negative | HFRT+NIC | non-pCR | pMMR | 2 |
| 7 | 48 | female | lower | 4.60 | IIIA(T2N1M0) | negative | positive | HFRT+NIC | pCR | pMMR | 3 |
| 8 | 70 | male | middle | 5.40 | IIIB(T3N2aM0) | negative | negative | HFRT+NIC | non-pCR | pMMR | 3 |
| 9 | 55 | male | lower | 2.40 | IIIB(T3N1M0) | negative | negative | HFRT+NIC | non-pCR | pMMR | 2 |
| 10 | 68 | female | middle | 3.80 | IIIB(T3N2aM0) | positive | positive | HFRT+NIC | pCR | pMMR | 2 |
| 11 | 77 | male | lower | 4.40 | IIIB(T3N2aM0) | negative | negative | HFRT+NIC | pCR | pMMR | 3 |
| 12 | 50 | female | middle | 5.20 | IIA(T3N0M0) | negative | positive | HFRT+NIC | non-pCR | pMMR | 3 |
| 13 | 62 | male | lower | 3.00 | IIIB(T3N1M0) | negative | negative | HFRT+NIC | non-pCR | pMMR | 3 |
| 14 | 76 | male | middle | 4.00 | IIIB(T3N2aM0) | positive | positive | HFRT+NIC | pCR | pMMR | 2 |
| 15 | 61 | female | lower | 5.60 | IIIB(T3N2aM0) | negative | negative | HFRT+NIC | pCR | pMMR | 2 |
| 16 | 59 | male | middle | 2.60 | IIIB(T3N1M0) | negative | negative | HFRT+NIC | non-pCR | pMMR | 3 |
| 17 | 75 | female | lower | 3.40 | IIIB(T3N1M0) | negative | positive | HFRT+NIC | non-pCR | pMMR | 3 |
| 18 | 45 | male | middle | 4.70 | IIIB(T3N2aM0) | positive | negative | HFRT+NIC | pCR | pMMR | 2 |
| 19 | 67 | female | lower | 5.80 | IIA(T3N0M0) | negative | positive | HFRT+NIC | pCR | pMMR | 1 |
| 20 | 73 | male | middle | 7.20 | IIIB(T4N1M0) | negative | negative | HFRT+NIC | non-pCR | pMMR | 3 |
| 21 | 52 | female | lower | 3.90 | IIA(T3N0M0) | negative | positive | HFRT+NIC | non-pCR | pMMR | 2 |
| **Patient ID** | **Ages (Years)** | **Sex** | **Tumor location** | **Length of tumor lesion (cm)** | **Clinical TNM stage** | **EMVI** | **MRI-CRM** | **Treatment** | **Pathological remission** | **MMR status** | **mrTRG** |
| 22 | 69 | male | middle | 4.30 | IIIB(T3N1M0) | positive | negative | HFRT+NIC | pCR | pMMR | 2 |
| 23 | 78 | female | lower | 5.70 | IIIB(T3N1M0) | negative | negative | HFRT+NIC | pCR | pMMR | 3 |
| 24 | 63 | male | middle | 2.90 | IIA(T3N0M0) | negative | positive | HFRT+NIC | non-pCR | pMMR | 4 |
| 25 | 56 | male | lower | 3.70 | IIIB(T3N1M0) | negative | negative | HFRT+NIC | pCR | pMMR | 2 |
| 26 | 71 | female | middle | 4.50 | IIIC(T4aN2aM0) | positive | positive | HFRT+NIC | non-pCR | pMMR | 3 |
| 27 | 64 | male | lower | 5.30 | IIIB(T3N2aM0) | negative | negative | HFRT+NIC | pCR | pMMR | 2 |
| 28 | 57 | female | middle | 2.70 | IIIB(T3N1M0) | negative | negative | HFRT+NIC | non-pCR | pMMR | 2 |
| 29 | 58 | male | lower | 3.30 | IIIB(T3N1M0) | negative | positive | HFRT+NIC | non-pCR | pMMR | 3 |
| 30 | 66 | male | middle | 4.10 | IIIC(T3N2bM0) | positive | negative | HFRT+NIC | pCR | pMMR | 1 |
| 31 | 63 | male | lower | 4.00 | IIIB(T3N1M0) | negative | positive | HFRT+NIC | pCR | pMMR | 2 |
| 32 | 71 | male | lower | 5.00 | IIIB(T3N1M0) | positive | negative | HFRT+NIC | non-pCR | pMMR | 3 |
| 33 | 46 | female | middle | 3.00 | IIIB(T3N1M0) | negative | negative | HFRT+NIC | pCR | pMMR | 2 |
| 34 | 59 | male | lower | 4.20 | IIA(T3N0M0) | positive | positive | HFRT+NIC | non-pCR | pMMR | 3 |
| 35 | 53 | male | middle | 2.20 | IIIB(T3N1M0) | positive | negative | HFRT+NIC | non-pCR | pMMR | 3 |
| 36 | 61 | male | lower | 4.80 | IIIB(T3N2aM0) | negative | positive | HFRT+NIC | pCR | pMMR | 1 |
| 37 | 53 | female | middle | 2.80 | IIA(T3N0M0) | positive | negative | HFRT+NIC | non-pCR | pMMR | 2 |
| 38 | 49 | male | lower | 5.20 | IIIB(T3N2aM0) | negative | negative | HFRT+NIC | non-pCR | pMMR | 2 |
| 39 | 45 | female | middle | 3.60 | IIIC(T4N2aM0) | positive | positive | HFRT+NIC | pCR | pMMR | 1 |
| 40 | 60 | male | lower | 4.60 | IIIB(T3N2aM0) | negative | negative | HFRT+NIC | pCR | pMMR | 2 |

Abbreviation: EMVI: Extramural Vascular Invasion; CRM: Circumferential Resection Margin; HFRT: Hypofractionated Radiotherapy; NIC: Neoadjuvant immunochemotherapy.

**Table2. Clinical characteristics according to the pathological remission of patients involved in the predict cohort.**

| **Characteristics** | **pCR** | **non-pCR** | **Significance** |
| --- | --- | --- | --- |
|  | **n=20 (%)** | **n=20(%)** |  |
| Age(years) | 61.40 ± 10.17 | 61.00 ± 8.27 | 0.217 |
| Gender |  |  | 0.393 |
| Male | 8 (40) | 13 (65) |  |
| Female | 12 (60) | 7 (35) |  |
| Length of tumor lesion (cm) | 4.59 ± 1.18 | 3.89 ± 1.30 | 0.718 |
| Tumor location |  |  |  |
| Middle | 8 (40) | 11 (55) |  |
| Lower | 12 (60) | 9 (45) |  |
| Clinical TNM stage |  |  | 0.972 |
| II | 3 (15) | 6 (30) |  |
| III | 17 (85) | 14 (70) |  |
| MRI-CRM |  |  | 0.544 |
| Negative | 12 (60) | 11 (55) |  |
| Positive | 8 (40) | 9 (45) |  |
| EMVI |  |  | 0.705 |
| Negative | 14 (70) | 13 (65) |  |
| Positive | 6 (30) | 7 (35) |  |
| ISG15^+^MHC-I^+^ Neutrophils |  |  | **0.029**** |
| Elevated after HRT | 13 (65) | 5 (25) |  |
| Decrease after HRT | 7 (35) | 15 (75) |  |
| mrTRG |  |  | **0.012**** |
| 1+2 | 15 (75) | 6 (30) |  |
| 3+4 | 5 (25) | 14 (70) |  |

The p-values for categorical variables were analyzed by Chi-square test and Fisher’s exact test, and continuous variables were analyzed by two independent-samples t-test. **p<0.01.

Abbreviation: EMVI: Extramural Vascular Invasion; CRM: Circumferential Resection Margin; HRT: Hypofractionated Radiotherapy.

**Table S3 Real-time quantity PCR primers sequences and Chip-PCR sequences**

| **Gene** |  | **Sequence (5'-3')** |
| --- | --- | --- |
| *mouse-GAPDH* | F  R | GGTGAAGGTCGGTGTGACCG  CTCGCTCCTGGAAGATGGTG |
| *mouse-H2-K* | F  R | CAGGTGGAGCCCGAGTATTG  CGTACATCCGTTGGAACGTG |
| *mouse-H2-D* | F  R | ACCACACATTCCGGCACAC  GTCCTCGTCATAGGTCTCCG |
| *mouse-H2-A* | F  R | GACCACGTAGGCACCTATGG  CTACAGCTATGTTTTGCAGTCCA |
| *mouse-H2-E* | F  R | GACCCCTCCGGTGATTTTAGT  CACACGCTCCTTCCCATTGA |
| *mouse-CTSA* | F  R | CAGCCCTCTTTCCGGCAATA  TTTGGGTCGTTCTGCGACTC |
| *mouse-CTSB* | F  R | CAGGCTGGACGCAACTTCTAC  TCACCGAACGCAACCCTTC |
| *mouse-CTSC* | F  R | GTTCCCGAAGCGACATTAACT  TCGTAGGCAGTATCCAACTTCTT |
| *mouse-CTSD* | F  R | GCTTCCGGTCTTTGACAACCT  CACCAAGCATTAGTTCTCCTCC |
| *Human-HLA-A*  *(Site 1)* | F  R | AAGGCGGTGTATGGATTG  CTGATTGGCTTCTCTGGAA |
| *Human-HLA-A*  *(Site 2)* | F  R | CAGTGGCTACTTTGTATTGAGGA  AATCTTGGTAACCCCTGAATG |

| **REAGENT or RESOURCE** | **SOURCE** | **IDENTIFIER** |
| --- | --- | --- |
| **Software and algorithms** | | |
| R version 4.3.1 | R Foundation | <http://www.R-project.org;> RRID:SCR_023486 |
| CellRanger version 4.0.0 | 10x Genomics | <https://www.10xgenomics.com/support;> RRID: SCR_023672 |
| Prism version 8.0 | GraphPad | <https://www.graphpad.com/;> RRID:SCR_002798 |
| FlowJo version 10.8.1 | BD Biosciences | <https://www.flowjo.com/;> RRID:SCR_000410 |
| SOAPnuke version 1.4.0 | BGI Genomics | <https://github.com/BGI-flexlab/SOAPnuke;> RRID:SCR_015025 |
| HISAT version 2.1.0 | BGI Genomics | <http://ccb.jhu.edu/software/hisat2/index.shtml;> RRID:SCR_015530 |
| Bowtie2 version 2.2.5 | BGI Genomics | <http://bowtie-bio.sourceforge.net/bowtie2/index.shtml;> RRID:SCR_016368 |
| RSEM version 1.2.8 | BGI Genomics | <http://deweylab.biostat.wisc.edu/rsem/;> RRID:SCR_000262 |
| DESeq version 1.39.0 | Bioconductor | <http://bioconductor.org/packages/release/bioc/html/DESeq.html;> RRID:SCR_000154 |
| ggplot2 version 3.4.4 | CRAN | <https://cran.r-project.org/web/packages/ggplot2/index.html;> RRID:SCR_014601 |
| Seurat version 4.3.0 | CRAN | <https://cran.r-project.org/web/packages/Seurat/index.html;> RRID:SCR_007322 |
| clusterProfiler version 4.12.6 | Bioconductor | <http://bioconductor.org/packages/release/bioc/html/clusterProfiler.html;> RRID:SCR_016884 |
| org.Hs.eg.db version 3.19.1 | Bioconductor | <https://bioconductor.org/packages/org.Hs.eg.db/;> RRID:SCR_024739 |
| Monocle version 2.32.0 | Github | <http://cole-trapnell-lab.github.io/monocle-release/docs/;> RRID:SCR_016339 |
| Monocle3 version 1.3.1 | Github | <https://cole-trapnell-lab.github.io/monocle3/;> RRID:SCR_018685 |
| CellChat version 1.6.1 | Github | <https://github.com/sqjin/CellChat;> RRID:SCR_021946 |
| Harmony version 1.2.1 | Github | <https://github.com/immunogenomics/harmony;> RRID: SCR_023543 |
| SingleR version 2.6.0 | Bioconductor | <https://www.bioconductor.org/packages/release/bioc/html/SingleR.html;> RRID:SCR_023120 |
| Slingshot version 2.12.0 | Github | <https://github.com/kstreet13/slingshot;> RRID:SCR_017012 |
| Cytotrace2 version 1.0.0 | Github | [https://github.com/digitalcytometry/cytotrace2](https://github.com/digitalcytometry/cytotrace2;) |
| miloR version 2.0.0 | Github | <https://github.com/MarioniLab/miloR;> RRID:SCR_025630 |
| scRNAtoolVis version 0.0.7 | Github | <https://github.com/junjunlab/scRNAtoolVis;>  RRID:SCR_023916 |
| ggrepel version 0.9.5 | CRAN | <https://cran.r-project.org/package=ggrepel;>  RRID:SCR_017393 |
| **Antibodies** | | |
| TruStain FcX anti-mouse CD16/32 (clone 93) | Biolegend | Cat# 101319; RRID: AB_1574973 |
| Anti-mouse CD45 (clone 30-F11) | Biolegend | Cat# 103126; RRID: AB_493535 |
| Anti-mouse Ly6G (clone 1A8) | Biolegend | Cat# 127643; RRID: AB_2565971 |
| Anti-mouse CD3 (clone 17A2) | Biolegend | Cat# 100236; RRID: AB_2561456 |
| Anti-mouse CD4 (clone RM4-5) | BD Biosciences | Cat# 553051; RRID: AB_398528 |
| Anti-mouse CD8a (clone 53-6.7) | Biolegend | Cat# 100730; RRID: AB_493703 |
| Anti-mouse IFN-γ (clone XMG1.2) | Biolegend | Cat# 505830; RRID: AB_2563105 |
| Purified anti-mouse CD3ε Antibody (clone 145-2C11) | Biolegend | Cat# 100301; RRID: AB_312666 |
| Purified anti-mouse CD28 Antibody (clone 37.51) | Biolegend | Cat# 102101; RRID: AB_312866 |
| PerCP/Cyanine5.5 anti-mouse H-2Kb/H-2Db Antibody | Biolegend | Cat# 114620; RRID: AB_2750200 |
| PE anti-human HLA-A,B,C Antibody | Biolegend | Cat# 311405; RRID: AB_314874 |
| PerCP/Cyanine5.5 anti-human CD66b | Biolegend | Cat# 305107; RRID: AB_2077856 |
| PE/Dazzle™ 594 anti-mouse H-2Kb bound to SIINFEKL Antibody | Biolegend | Cat# 141612; RRID: AB_2750522 |
| APC anti-human CD86 Antibody | Biolegend | Cat# 374208; RRID: AB_2721449 |
| Rabbit ISG15 Monoclonal antibody | Abcam | Cat# ab285367, RRID: AB_3712098 |
| Rabbit NOD1 Monoclonal antibody | Abcam | Cat# ab189409 |
| Rabbit MHC class I Monoclonal antibody | Abcam | Cat# ab134189, RRID: AB_3073854 |
| Rabbit JAK1 Monoclonal antibody | Abcam | Cat# ab324683 |
| Rabbit NF-kB p65 Monoclonal antibody | Abcam | Cat# ab32536, RRID: AB_776751 |
| Rabbit NF-kB p65 (phospho S468) Monoclonal antibody | Abcam | Cat# ab264271 |
| Rabbit GAPDH Monoclonal antibody | Abcam | Cat# ab8245, RRID: AB_2107448 |
| Alexa Fluor® 488-conjugated Goat Anti-Rabbit IgG (H+L) | ServiceBio | Cat# GB25303, RRID: AB_2910224 |
| Rabbit monoclonal [EPR28318-62] to ISG15 | Abcam | Cat# ab315281 |
| Recombinant Anti-CEACAM5 + CD66b + CEACAM1 + CEACAM6 antibody | Abcam | Cat# ab207718, RRID: AB_3086740 |
| **Biological samples** | | |
| Human LARC tissues | Cancer Center, Union Hospital, Tongji Medical College, Huazhong University of Science and Technology | N/A |
| **Chemicals, peptides, and recombinant proteins** | | |
| Fetal Bovine Serum (FBS) | Solarbio | Cat# S9030 |
| DMEM medium | Gibco | Cat# 11965092 |
| RPMI1640 medium | Gibco | Cat# 11875119 |
| Penicillin-streptomycin | Solarbio | Cat# P1400 |
| TRIzol | Invitrogen | Cat# 15596018CN |
| β-mercaptoethanol | Genom Bio | Cat# GNM21985-1 |
| Hyaluronidase | G-CLONE | Cat# 37326-33-3 |
| DNase I | G-CLONE | Cat# 9003-98-9 |
| Collagenase V | G-CLONE | Cat# 9001-12-1 |
| Intracellular Staining Permeabilization Wash Buffer (10X) | Biolegend | Cat# 421002 |
| Interleukin (IL)-2 | MedChemExpress | Cat# HY-P70646AF |
| Ionomycin | MedChemExpress | Cat# HY-13434 |
| Monensin sodium | MedChemExpress | Cat# HY-N0150 |
| Phorbol 12-myristate 13-acetate (PMA) | MedChemExpress | Cat# HY-18739 |
| InVivo MAb anti-mouse PD-1 | BioXcell | Cat# BE0146 |
| HiScript III RT SuperMix for qPCR (+gDNA wiper) | Vazyme | Cat# R323-01 |
| ChamQ SYBR qPCR Master Mix | Vazyme | Cat# Q311-02 |
| Triton X-100 Surfact-Amps | Thermo Fisher Scientific | Cat# 85111 |
| Reactive Oxygen Species Assay Kit | Beyotime | Cat# S0033S |
| Cell Counting Kit-8 | Beyotime | Cat# C0037 |
| Click-iT Edu-488 cell proliferation detection kit | Servicebio | G1601 |
| IFN alpha-IFNAR-IN-1 hydrochloride | MedChemExpress | Cat# HY-12836A |
| SARS-CoV-2 PLpro | MedChemExpress | Cat# HY-P70122 |
| NF-κB-IN-1 | MedChemExpress | Cat# HY-138537 |
| IFN-alpha 1/IFNA, Mouse | MedChemExpress | Cat# HY-P701053 |
| IFN-alpha 1/IFNA13, Human | MedChemExpress | Cat# HY-P70241 |
| NOD1-IN-1 | MedChemExpress | Cat# HY-168023 |
| JAK1-IN-13 | MedChemExpress | Cat# HY-161015 |
| OVA Peptide (257-264) | MedChemExpress | Cat# HY-P1489 |
| FAM-OVA (257-264) | MedChemExpress | Cat# HY-P1489F |
| **Critical commercial assays** |  |  |
| RNA 6000 Nano Kit | Agilent | Cat# 5067-1511 |
| Tumor Dissociation Kit | Miltenyi Biotec | Cat# 130-095-929; RRID: SCR_020276 |
| Dead Cell Removal Kit | Miltenyi Biotec | Cat# 130-090-101 |
| Chromium Single Cell 3’ Reagent Kit v3 | 10x Genomics | N/A |
| MojoSort Mouse CD8^+^ T Cell Isolation Kit | Biolegend | Cat# 480007 |
| MojoSort Mouse Neutrophil Isolation Kit | Biolegend | Cat# 480058 |
| Zombie NIR Fixable Viability Kit | Biolegend | Cat# 423105 |
| CFSE Cell Division Tracker Kit | Biolegend | Cat# 423801 |
| Human IFN alpha ELISA Kit | Invitrogen | Cat# BMS216, RRID: AB_2575462 |
| Human IFN beta ELISA Kit | Invitrogen | Cat# 414101 |
| Human IFN gamma ELISA Kit | Invitrogen | Cat# KHC4021 |
| **Deposited data** |  |  |
| Single cell RNA-seq data of tumor tissues in LARC patients | This paper | GEO: GSE278406 and GSE316027 |
| TCGA pan-cancer cohorts  (BRCA, GBM, COAD, LIHC, READ, ESCA) | https://portal.gdc.cancer.gov/ | N/A |
| **Experimental models: Cell lines** |  |  |
| Mouse MC38 | National Infrastructure of Cell-line Resource | 1101MOU-PUMC000523 |
| Mouse MC38-OVA | Shanghai hexu biology | Cat# HX-0349; RRID: CVCL_XJ96 |
| HL60 | National Infrastructure of Cell-line Resource | RRID: CVCL_2944 |
| Primary CD8^+^ T cell from speen of C57BL/6J mice | This paper | N/A |
| Primary neutrophils cell from bone marrow of C57BL/6J mice | This paper | N/A |
| **Experimental models: Organisms/strains** |  |  |
| Mouse: C57BL/6J (Male, 6- to 7-week-old) | WUHAN MOUBAILI BIOTECHNOLOGY Co., Ltd | RRID: MGI:3028467 |
| Mouse: OT-1 | WUHAN MOUBAILI BIOTECHNOLOGY Co., Ltd | N/A |
| **Oligonucleotides** |  |  |
| Primers for RT-qPCR | Table S3 | N/A |
| Chip-PCR sequences | Table S3 | N/A |
| **Other** |  |  |
| 2100 Bioanalyzer | Agilent | N/A |
| Dr.Tom online platform | BGI Genomics | https://biosys.bgi.com |
| NovaSeq 6000 Sequencing System | Illumina | <https://www.illumina.com/systems/sequencing-platforms/novaseq.html;>  RRID:SCR_016387 |
| CytoFLEX flow cytometer | Beckman Kurt Trading Co., Ltd | N/A |
| StepOnePlus real-time PCR system | Thermo Fisher Scientific | Cat# 4376600 |
| The Human Protein Atlas | SciCrunch Registry | <http://www.proteinatlas.org/> RRID:SCR_006710 |
| JASPAR | Centre for Molecular Medicine Norway | [http://jaspar.genereg.net](http://jaspar.genereg.net/)  RRID:SCR_003030 |
| ChIP-Atlas | RIKEN | <http://chip-atlas.org/>  RRID:SCR_015511 |
| National Center for Biotechnology Information | NIH | [http://www.ncbi.nlm.nih.gov](http://www.ncbi.nlm.nih.gov/)  RRID:SCR_006472 |
